# Incentives are effective but insufficient to halt smallholder deforestation in the Colombian Amazon

**DOI:** 10.1371/journal.pone.0340539

**Published:** 2026-09-23

**Authors:** Hugo Charoud, Esteve Corbera, Lina Moros, Sébastien Costedoat, Santiago Izquierdo-Tort

**Affiliations:** 1 G-EAU, University of Montpellier, AgroParisTech, INRAE, Institut Agro, IRD, Montpellier, France; 2 Institució Catalana de Recerca i Estudis Avançats (ICREA), Barcelona, Spain; 3 Institute of Environmental Science and Technology, Universitat Autònoma de Barcelona, UAB, Cerdanyola del Vallès, Spain; 4 Department of Geography, Universitat Autònoma de Barcelona, UAB, Cerdanyola del Vallès, Spain; 5 Facultad de Administración, Universidad de los Andes, Bogotá, Colombia; 6 Moore Center for Science, Conservation International, Arlington, United States of America; 7 Department of Forest and Resources Management, Faculty of Forestry, The University of British Columbia, Vancouver, Canada; North Carolina State University, United States of America

## Abstract

In this article, we assess the impact of two Payments for Ecosystem Services (PES) initiatives aimed at deterring deforestation in the department of Guaviare, Colombia. The initiatives are the government-led *Incentivo Forestal Amazónico* (IFA), which is part of the country’s REDD+ strategy, and the NGO-driven *Caminemos–Territorios Sostenibles* (CTS) project, which supported sustainable land-use activities over a four-year period. We find that, combined, these initiatives prevented approximately 1,400 hectares of forest loss, though they differed in their annualized impacts (2.9% *versus* 3.8%). We argue that this difference likely reflects variations in the type of incentives provided, their geographic focus, and their overarching goals. IFA focuses on larger, less threatened forest patches across a broader area, providing cash payments to landowners whilst laying the foundations for a long-term conservation and management approach. CTS, in contrast, targeted smaller patches at higher risk of deforestation along Guaviare’s agricultural frontier, offering targeted technical assistance and payments for a series of conservation and restoration commitments at farm level, including the adoption of silvopastoral practices. Considering these findings, we argue that distinct PES designs can play a complementary role in halting deforestation in the Colombian Amazon, and potentially in similar regions across tropical America. We call for more sustained and scaled-up efforts in sustainable land use and forest conservation for smallholders, while recognizing that such initiatives will remain insufficient unless scaling is accompanied by substantially stronger territorial state presence and governance in areas contested by armed groups, as well as comprehensive land-use policy reform.

## Introduction

Deforestation is one of the most pressing environmental challenges of our time. It contributes to biodiversity loss and represents the main driver of land-use related emissions, at around 3.9 GtCO2 per year over the 2015–2024 period [1]. Critically, tropical forests –which include much of the world’s biodiversity, sustain the livelihoods of diverse Indigenous and local communities, serve as important carbon sinks, and provide other essential ecosystem services– are being depleted, converted into pastures, agro-industrial crop fields, and human infrastructures [2,3].

Since the early 2000s, Payments for Ecosystem Services (PES) programs have been adopted globally as a key instrument to promote forest management, restoration, and conservation. PES consist of monetary -and sometimes also in-kind- incentives provided to landowners and communities in the form of short-term contracts (i.e., 3–5 years), conditional on undertaking certain activities that should protect standing forests or enhance the provision of ecosystem services. They have gained popularity because they can provide both conservation and socio-economic benefits [4] and, as of today, there are hundreds of PES programs and projects distributing dozens of billions of dollars in annual transactions [5]. These PES programs vary widely in their design and implementation aspects, including their goals –ecosystem conservation or provision of specific ecosystem services (e.g., carbon, water, biodiversity)–, geographic scale –local, subnational, national, international–, participating actors –governments, NGOs, private companies, multilateral organizations–, and contract design features –e.g., incentive structure, targeting, enforcement– [6].

A key innovation of PES has been their conditionality and voluntary character, which reduced concerns about lack of retribution and exclusion that were common in prior command-and-control instruments such as protected areas [7]. Directly linking the provided incentives to specific outcomes or land-use activities is also expected to address common shortcomings of earlier, more indirect approaches like Integrated Conservation and Development Projects (ICDPs) [8]. Cash payments or in-kind benefits like seedlings, equipment, and training can help increase the competitiveness of forest conservation relative to alternative land uses that are more economically profitable in the absence of PES [9].

A growing body of research has focused on assessing PES effectiveness and the factors that explain their success or failure. PES can reduce deforestation through several mechanisms that can act synergistically: by increasing the relative returns to forest conservation compared to alternative land uses, by encouraging the adoption of low-deforestation productive practices, and by strengthening land-use planning, collective environmental governance, monitoring, and compliance. PES are expected to change land-use behavior by increasing the financial returns of conservation, supporting the intensification of more sustainable agriculture, or strengthening local conservation institutions. However, as noted earlier, these mechanisms do not always operate in conjunction or perform as intended. Their effects may be undermined by high opportunity costs of forest conservation, weak enforcement and insecure land tenure, or strong pressures from external factors, such as cattle ranching, agro-industrial practices, or illegal economies.

Results from impact evaluations of PES ecological benefits -which is the outcome of concern in this article- show that large-scale programs, for example in Mexico or Costa Rica, have generated positive but modest reductions in deforestation at the national level [10]. Evidence of PES effectiveness at the local level is mixed, with results ranging from highly positive [11,12] to neutral and even negative [13–15]. The latter has been often related to targeting areas with low deforestation risk [16], while greater success has been observed when PES programs focus on high-risk frontier areas and engage smallholder communities [17]. The degree of PES ecological effectiveness has also been related to land tenure security and the strength of conservation-oriented institutions [7], the monitoring of compliance levels and sanctioning practices [6], and by strategic program adoption among participants [18,19].

In this article, we analyze the success of two PES initiatives in achieving their goal of deterring deforestation in the department of Guaviare, Colombia. The *Incentivo Forestal Amazónico* (IFA) program provides monetary payments for forest conservation and is implemented by the Colombian government under the broader policy framework of *Visión Amazonía*, an ambitious REDD+ Early Movers program funded by the governments of Norway, the United Kingdom, and Germany (https://visionamazonia.minambiente.gov.co/). Launched in 2018, the program is jointly managed by the NGO Más Bosques, three environmental authorities of the Colombian Amazon region, and the Ministry of Environment and Sustainable Development, each assuming distinct roles. IFA is a key pillar of the REDD+ strategy and operates as a conventional PES intervention across five departments of the country’s Amazon region (Guaviare, Caquetá, Putumayo, Meta, Amazonas), where direct cash payments are provided to households in exchange for conservation commitments over clearly defined forest areas. The *Caminemos Territorios Sostenibles* (CTS) project, in turn, was implemented between 2018 and 2022 by the NGO ONF-Andina along the agricultural frontier of Guaviare. It combined both cash and in-kind land-use planning technical assistance, with the former being tied to the amount and type of conservation and restoration activities to be performed on the farm (see *Materials and methods*).

For both IFA and CTS, we specifically ask: how do government-led REDD+ payments and NGO-provided incentives contribute to reducing deforestation? How do distinct design and implementation features affect avoided deforestation outcomes? Understanding the effectiveness of current PES policies and projects is particularly urgent in a region where new actors are grabbing forest land for cattle ranching, coca cultivation, and other extractive activities such as gold mining [20–23]. It is also important to examine the differing impacts of two contrasting PES designs: one focused on payments only and broad in reach with another providing land-use planning assistance as well as economic support in a more circumscribed area of the agricultural frontier. Comparing these approaches can provide important insights into what works best and why for reducing deforestation in the study region.

To our knowledge, only one study has assessed the ecological effectiveness of PES in the Colombian Amazon [24]. Three additional studies have examined environmental outcomes of PES in Colombia, although all are limited to silvopastoral PES schemes in the Quindío and Valle del Cauca regions [25–27]. None addresses the two gaps we seek to fill in this article, i.e., how well two PES programs which overlap in the same department perform and, how such performance is influenced by the programs’ design and implementation features. Elsewhere, research has explored how different conservation and social policy instruments interact within the same landscape, for example protected areas and PES [10,28,29] or PES and anti-poverty programs [30]. In tropical frontier regions like Guaviare, where deforestation pressure is acute, it is common for multiple conservation projects led by public agencies, civil society, and private actors to coexist [31]. While this proliferation of environmental programs can help fill governance gaps and mobilize rapid action, it can also create inefficiencies and coordination challenges [32]. Analyzing the overlapping but contrasting designs of IFA and CTS therefore enables us to assess the combined effects of policy mixes and to compare how differences in incentive structure, conditionality, and governance arrangements influence conservation outcomes. Addressing these interactions is especially important considering emerging international frameworks, such as the Tropical Forest Forever Facility, which aim to scale up incentive-based forest conservation in complex, multi-actor settings.

## Materials and methods

### Analytical framework

To analyse the ability of IFA and CTS in halting deforestation, we employed a causal inference approach, combining propensity reweighting and a generalized Difference-in-Difference (DiD) model with covariates’ matching. Causal inference has been used extensively in conservation impact evaluation, to explicitly address the selection bias inherent in programs based on voluntary or selective participation [12]. It is accepted that one can estimate the Average Treatment Effect on the Treated (ATT) by using the outcome of a group of non-treated units as a counterfactual for the outcome of a group of treated units (ibid.). In this context, DiD models aim to replicate a natural experiment in which the outcome of treated and non-treated unit groups over at least two periods can be compared, e.g., before and after the implementation of the treatment. The non-treated group is used as a control, and one can subtract the outcome gains of the non-treated from the gains of the treated group. This is theoretically possible because DiD models rest on the so-called Conditional Parallel Trends Assumption (CPTA) which states that the treated and control groups would have followed the same path over time if the treatment had not occurred. This assumption permits to isolate the causal effect of the treatment by comparing the actual outcome of the treated group to the counterfactual trend of the control group.

### Geographical context and characterization of the studied initiatives

Six percent of the Amazon biome is in Colombia, covering an area of 48.3 million hectares, i.e., 42% of the country’s total land area. The establishment of protected areas and the presence of guerrilla groups since the late 1960s, the government’s recognition in the 1991 Constitution of Indigenous Peoples’ territories (known as *Resguardos Indígenas*) and the creation of the National Environmental System in 1993 helped keeping deforestation rates relatively low across Colombia’s Amazon until the early 2000s. However, the last two decades have witnessed a rapid increase in deforestation in the region and beyond, driven by legal and illegal land-use activities [33,34]. In 2017, the country experienced a historic peak of deforestation (219,000 hectares, 71% in the Amazon), because of the governance void left by the dismantling of the principal guerrilla groups. This trend also explains why almost 40% the country’s greenhouse gas emissions originate in the land use, land use change and forestry sector [35].

The department of Guaviare is one of the country’s poorest [36], with a population of 56,758 inhabitants scattered around four municipalities and 12 Indigenous territories. Agriculture accounts for 12% of its GDP and employs 11% of the department’s labor force. The main income-generating activities are cattle grazing, followed by rubber, cocoa, and banana cultivation (ibid.), and 80% of the department’s land is still covered by tropical moist forest [37], which hosts a high concentration of species diversity and endemism [38]. The most populated town is the capital city, *San José del Guaviare*, located in the north at the intersection of three main rivers: the *Ariari*, the *Guayabero*, and the *San José*. The other three main towns (and their corresponding municipalities) lie farther south. *Calamar* and *El Retorno* are directly connected to *San José del Guaviare* via road 75, which begins in the departmental capital and ends in *Calamar*. These two municipalities were established more recently and are less densely populated. The municipality of *Miraflores* is situated even farther south and is the most isolated, as it is accessible only by unpaved roads. In addition to their main town centers, all municipalities include several dispersed rural settlements, known as *veredas*, which are scattered across the territory and often located along informal or semi-formal roads.

**Fig 1:**
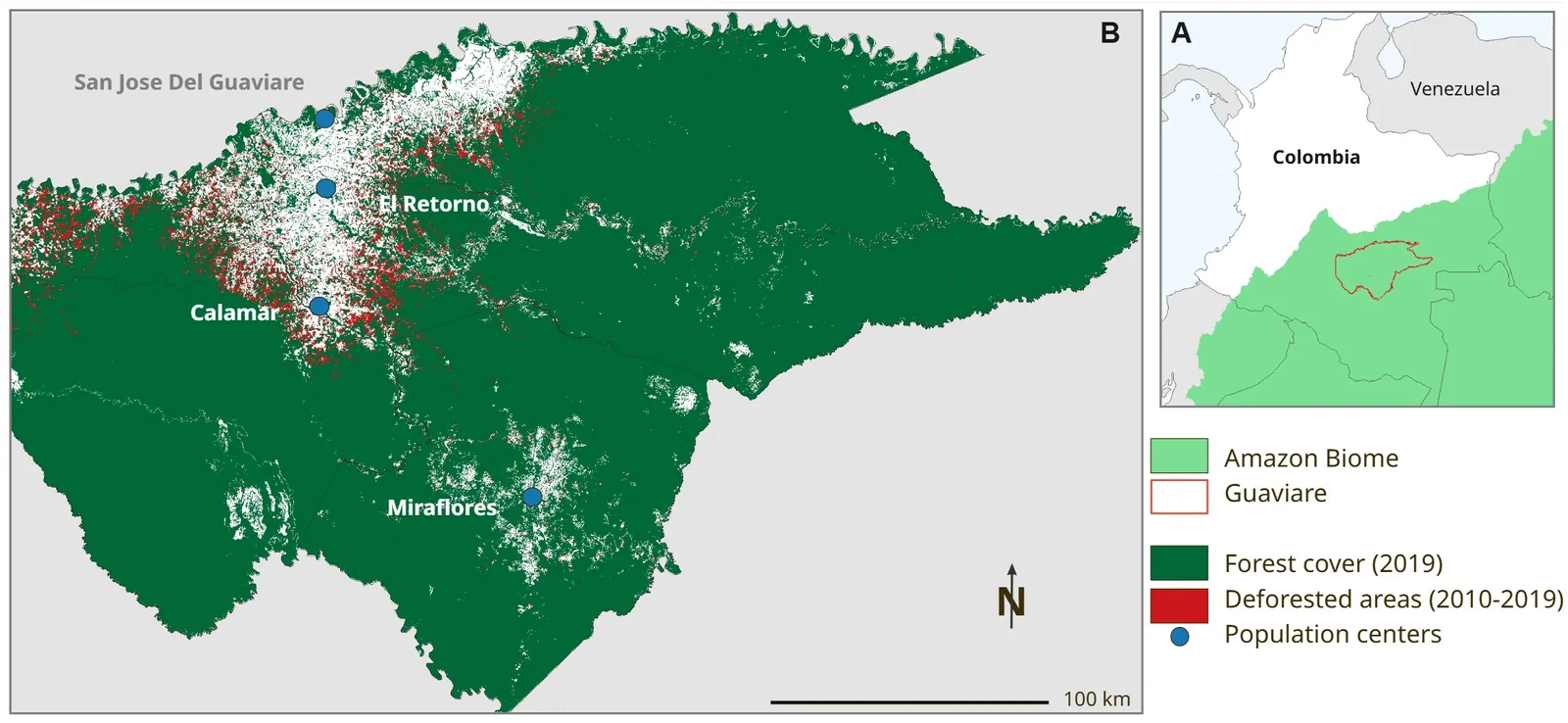
Recent deforestation in Guaviare, 2010-2019. Source: authors’ own elaboration using terra and sf packages in R studio, based on data from the European Commission Joint Research Centre Tropical Moist Forests observatory (JRC-TMF). Dataset available at https://ies-ows.jrc.ec.europa.eu/iforce/tmf_v1/download.py?type=tile&dataset=DeforestationYear&lat=N10&lon=W80 [Deforestation]. Disclaimer: The designations employed and the presentation of material on this map do not imply the expression of any opinion whatsoever on the part of the European Union concerning the legal status of any country, territory, city or area or of its authorities, or concerning the delimitation of its frontiers or boundaries.

**Fig 2:**
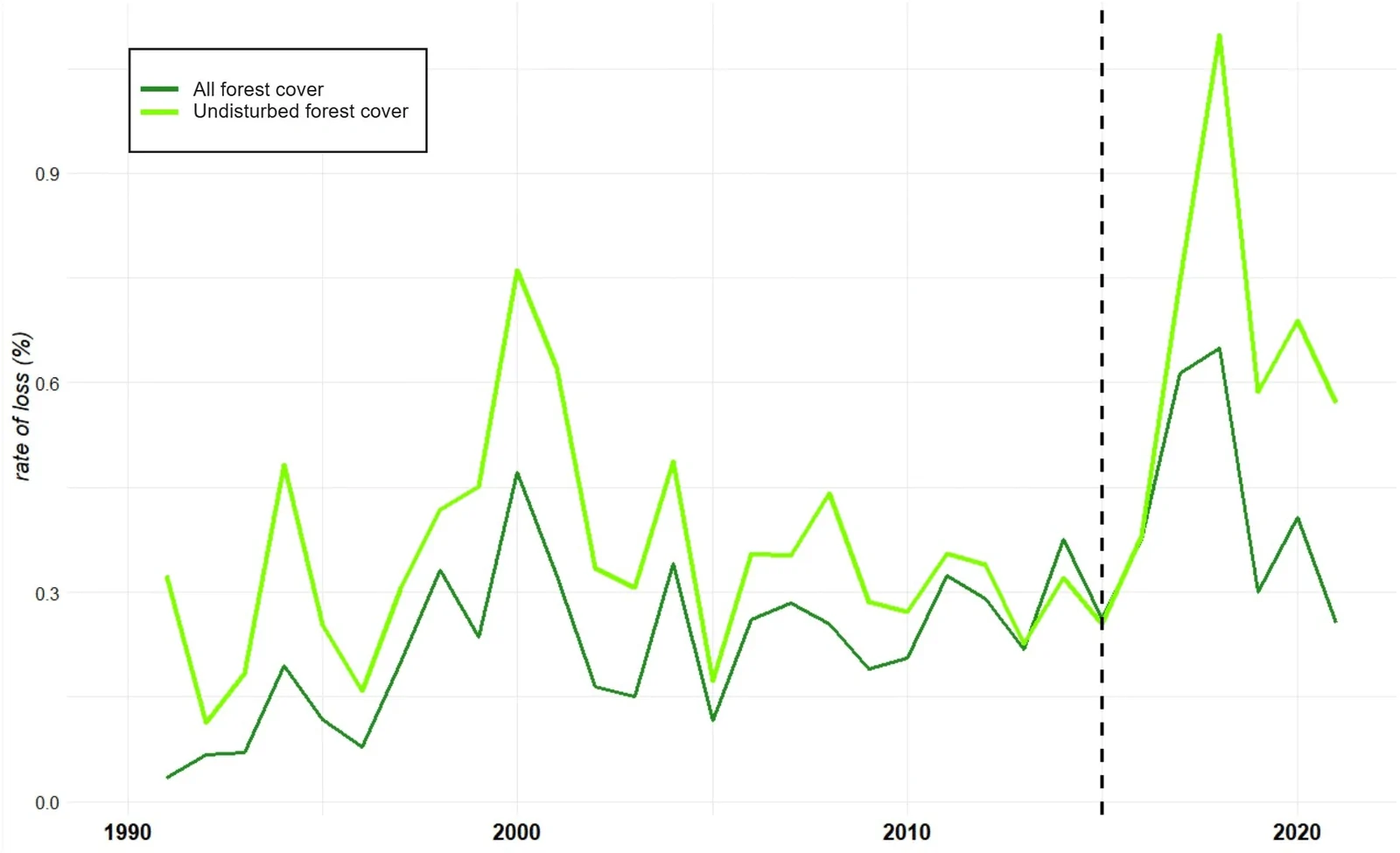
Evolution of the forest loss rate in Guaviare, 1991-2021. Source: authors’ own elaboration, based on data from the JRC-TMF. Datasets available at https://ies-ows.jrc.ec.europa.eu/iforce/tmf_v1/download.py?type=tile&dataset=DeforestationYear&lat=N10&lon=W80 [Deforestation], and https://ies-ows.jrc.ec.europa.eu/iforce/tmf_v1/download.py?type=tile&dataset=DegradationYear&lat=N10&lon=W80 [Degradation]. Caption: The dashed line indicates the year (2016) when the peace accords were signed. Undisturbed forest is defined as forest area for which no perturbation has been detected. All forest cover includes undisturbed forest cover and forest cover for which short-term perturbations have been detected.

**Fig 3:**
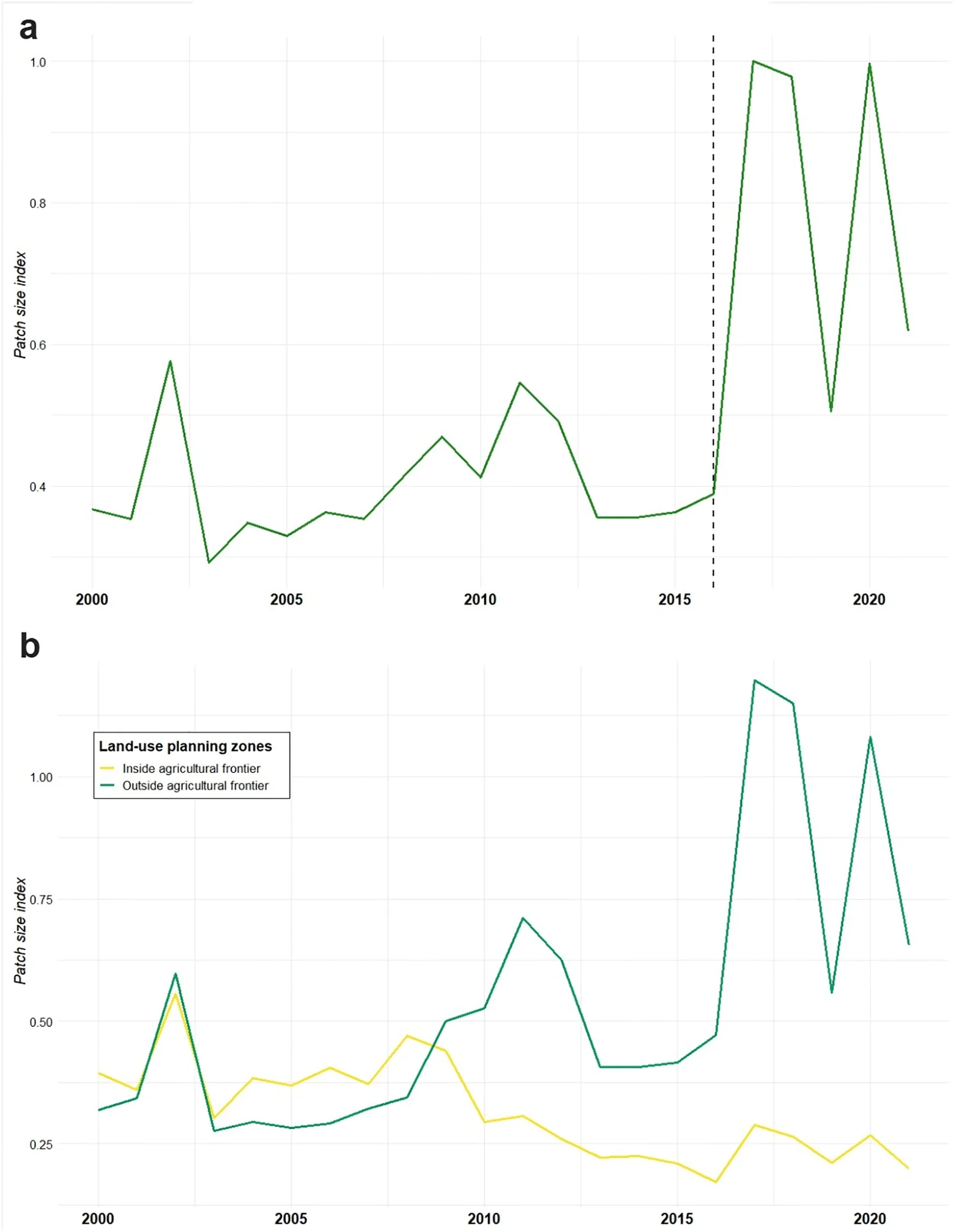
Evolution of the deforestation patch size index in Guaviare (panel a), inside or outside of the department’s agricultural frontier (panel b), 2000-2021. Source: authors’ own elaboration based on data from SIAT-AC. Dataset available at https://datos.siatac.co/pages/coberturas [Land-use cover 1:100.000]. Caption: the dotted line indicates the year (2016) when the peace accords were signed. The agricultural frontier (*Frontera Nacional Agrícola*) is a land-use planning zone defined by the Colombian Ministry of the Environment, encompassing areas where deforestation is not illegal and has been prevalent over the past ten years.

Deforestation and forest degradation are proceeding apace in Guaviare, which has lost 5.6% of its forest cover since the early 2000s [39]. This represents almost 60% of total forest loss in the country over these past two decades, with an expanding agricultural frontier along the northwest [34] and with a sharp increase since the signing of the peace accords in 2016 (Fig 1 and 2). As in other departments of the country’s Amazon, deforestation and forest degradation have been indirectly driven by coca cultivation and cattle grazing, which have contributed to assert land ownership across the tropics, often sparked by non-local stakeholders, particularly in remote areas [21,23]. Indeed, our analysis of land-use change in Guaviare using a deforestation patch index (see *Forest cover data, impact analysis area and matching covariates*) shows that the average size of converted landholdings increased after 2016 (Fig 3a). From then onwards, deforestation has been increasingly observed in more remote areas, including within Indigenous territories and protected areas where individual land ownership and land-use conversion are, in principle, prohibited (Fig 3b).

### The Incentivo Forestal Amazónico (IFA)

As noted in the Introduction, the IFA program aims to reduce deforestation by providing monetary incentives to smallholders for forest conservation. Between 2020 and 2022, it had enrolled 109,425 hectares of primary forest across its five targeted departments. In Guaviare, implementation in Guaviare began in 2020 and was scaled up from 2021 onwards. The pilot phase in 2020 included 194 participants; however, contracts lacked clearly defined conservation areas, payment rules were still under development, and compliance monitoring was weak. For these reasons, we excluded the pilot phase from our analysis. By early 2023, 516 households in 61 communities across the department’s four municipalities had joined IFA, enrolling landholdings for a total of 25,862 hectares and placing 16,615 hectares under forest conservation contracts (Table 1).

**Table 1:** Key characteristics of the studied programs in Guaviare.

	IFA	CTS
Contracts signed	516	400
Total area of the landholdings enrolled (ha)*	25,862	27,606
Total area of forest under conservation (ha)	16,615	12,301
Number of communities enrolled	61	40
Years of activity	2021-2022	2018-2022
Contract start date	June 2021	Between June 2018 and June 2019
Contract length	Annual (renewable)	From 3 to 4 and a half years
Minimum forested area of the landholding required to participate	30%	None
Type of benefits	Direct cash transfers	Technical assistance & financing of sustainable land-use activities
Range of payments per participant household	2.4-3.6 million COP (570–850 EUR – per year)	7−12 million COP (1600−2,800 EUR – over the whole contract period)
Key actors	Más Bosques, CDA, Ministry of the Environment and Sustainable Development (REDD + “Visión Amazonía”)	ONF-Andina
Funders	Norway, UK, Germany through the REDD+ Early Movers fund	European Union’s Fund for Peace

* For the impact analysis conducted in this research, we consider treated the overall area of the forest plots (IFA) / landholdings (CTS) registered, to account for on-site leakage (i.e., farmers displacing their deforestation to other parts of their property where conservation is not being incentivized).

To be eligible for enrolment, the smallholder’s property had to have at least 30% of its total area under forest cover and contain a minimum of 10 hectares of forest. This implied a minimum total property size of 33.3 hectares. This rule was established to encourage the participation of smaller plots, particularly in Putumayo, where average plot sizes are smaller than in Guaviare or Meta. The program aimed to enroll all forested areas within a family property landholding; however, for smaller properties, participants could freely decide how much forest to include in the contract, provided they met the 10-hectare minimum. The enrolled area determined the payment received: 2.4 million COP per year for enrolling 30–59% of the forested area, 3 million COP for 60–74%, and 3.6 million COP for more than 75%. Payments were delivered in four instalments via bank transfer, and contracts lasted approximately three years. Compliance was measured using satellite monitoring, considering the early deforestation alerts issued by IDEAM, and complemented with field visits, when possible.

### Caminemos Territorios Sostenibles (CTS)

The five-year CTS project implemented by ONF-Andina was aimed at developing sustainable land-use plans for family farms along the agricultural frontier of Guaviare, i.e., in the northwestern part of the department, to encourage on-farm conservation and restoration activities, as well as the conversion of extensive cattle grazing into a silvopastoral system. To be eligible for enrolment, each household had to characterize and map its landholding and develop the correspondent sustainable land-use plan. The technical and financial support provided depended on the ecological characteristics of the farm, the number of conservation and restoration activities to be implemented, and the investment and goods required (e.g., barbwire, seedlings, waterholes, etc.) to move towards a silvopastoral system. The NGO assigned each enrolled household a score ranging from 0 to 100, and the payment level depended on the household’s capacity to cover the costs of the planned activities, the degree of ambition and viability of such activities, the hectares of forests on the farm, and the household’s past deforestation behavior. As a result, payments per household oscillated between 7 and 12 million COP over the project’s timeframe. Compliance was monitored through CTS’s own platform, Forland, which used satellite imagery to generate monthly deforestation alerts [40]. A total of 400 participants across 40 communities enrolled gradually between June 2018 and June 2019, covering 27,606 hectares and placing 12,301 hectares of these under conservation. Each household was enrolled for approximately three to four years (Table 1).

### Targeted areas and deforestation risk

**Fig 4:**
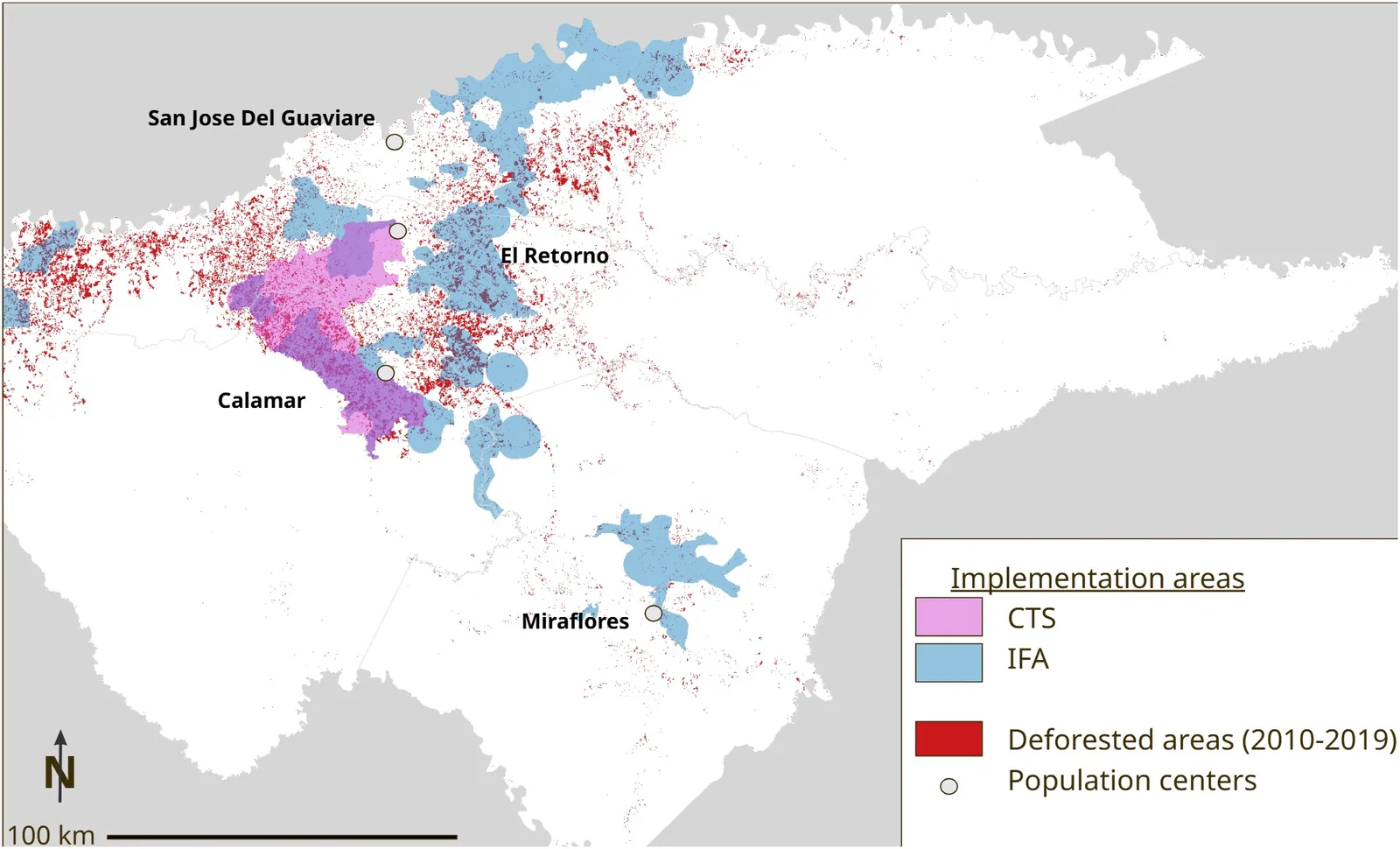
IFA and CTS implementation areas. Source: authors’ own elaboration using terra and sf packages in R studio, based on data from the JRC-TMF. Dataset available at https://ies-ows.jrc.ec.europa.eu/iforce/tmf_v1/download.py?type=tile&dataset=DeforestationYear&lat=N10&lon=W80 [Deforestation]. Disclaimer: The designations employed and the presentation of material on this map do not imply the expression of any opinion whatsoever on the part of the European Union concerning the legal status of any country, territory, city or area or of its authorities, or concerning the delimitation of its frontiers or boundaries.

The IFA program targets forests located along Guaviare’s deforestation arc as well as relatively intact forests in the municipality of Miraflores (Fig 4), whereas the CTS project focused solely on the area where the municipalities of *San José del Guaviare*, *El Retorno*, and *Calamar* intersect. IFA participants’ landholdings have, on average, 70% forest cover, although only 64% of that cover is enrolled in conservation contracts. In contrast, CTS participants’ landholdings had an average of 43% forest cover, with only 44% included in their land-use plans as a conservation long-term commitment. This confirms that the CTS project targeted more consolidated agricultural areas and that most of its participants had already deforested half or more of their landholdings. CTS participants’ enrolment of a lower proportion of their forests in the contracts may also suggest a higher willingness to engage in deforestation soon.

**Fig 5:**
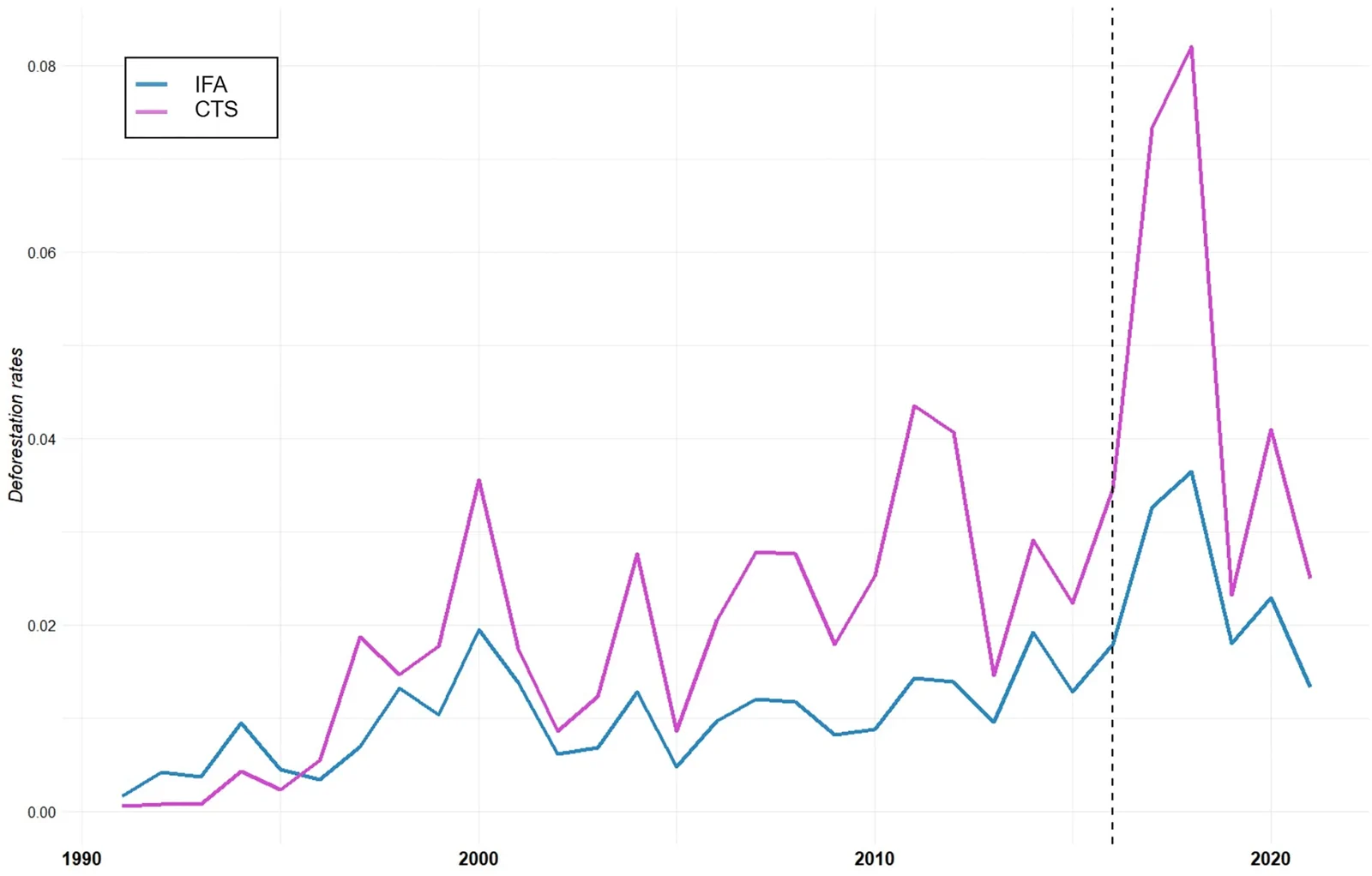
Evolution of the deforestation rate in IFA and CTS implementation areas, 1990-2022. Source: authors’ own elaboration based on data from the JRC-TMF. Dataset available at https://ies-ows.jrc.ec.europa.eu/iforce/tmf_v1/download.py?type=tile&dataset=DeforestationYear&lat=N10&lon=W80 [Deforestation].

Fig 5 illustrates the differences in average deforestation rates over the past 30 years across IFA and CTS implementation areas and shows that rates have been consistently higher in CTS areas. It is then not surprising that deforestation risk also differs between the areas targeted by the two initiatives: IFA-enrolled forest plots have, on average, steeper slopes and are located farther from river courses, roads, and urban centers or communities. These plots are also more likely to be situated away from the agricultural frontier and closer to national parks or forest reserves compared with CTS-enrolled landholdings (Table 2).

**Table 2:** Average mean values for key geographical variables of the IFA and CTS enrolled forest plots.

	IFA	CTS
Distance to roads (km)	2.43	1.13
Distance to San Jose (km)	69.8	56.3
Distance to municipality center (km)	34.2	21.1
Distance to natural parks (km)	11.3	26.5
Distance to rivers (km)	39.8	43.5
Elevation (m)	224	266
Slope	0.058	0.050

### Forest cover data, impact analysis area and matching covariates

Forest cover and land-use change data were obtained from i) the European Commission Joint Research Centre Tropical Moist Forests (JRC-TMF) dataset (https://forobs.jrc.ec.europa.eu/TMF), to track long-term changes in forest cover in the study region, and ii) the *Sistema de Información Ambiental Territorial de la Amazonia Colombiana* (SIAT-AC) database (https://siatac.co/) -hosted by the *Instituto Amazónico de Investigaciones Científicas* (SINCHI)- to develop the impact analysis. The former provides global forest-cover maps at 30-meter resolution since 1990 for all tropical moist forests, including the Colombian Amazon. It also distinguishes between deforestation and forest degradation. A pixel is classified as deforested if LANDSAT imagery detects perturbations lasting more than two consecutive years; if the perturbation lasts for a shorter period, it is categorized as forest degradation. Consequently, there is uncertainty regarding the nature of disturbances (deforestation or degradation) in the most recent two years of data.

**Fig 6:**
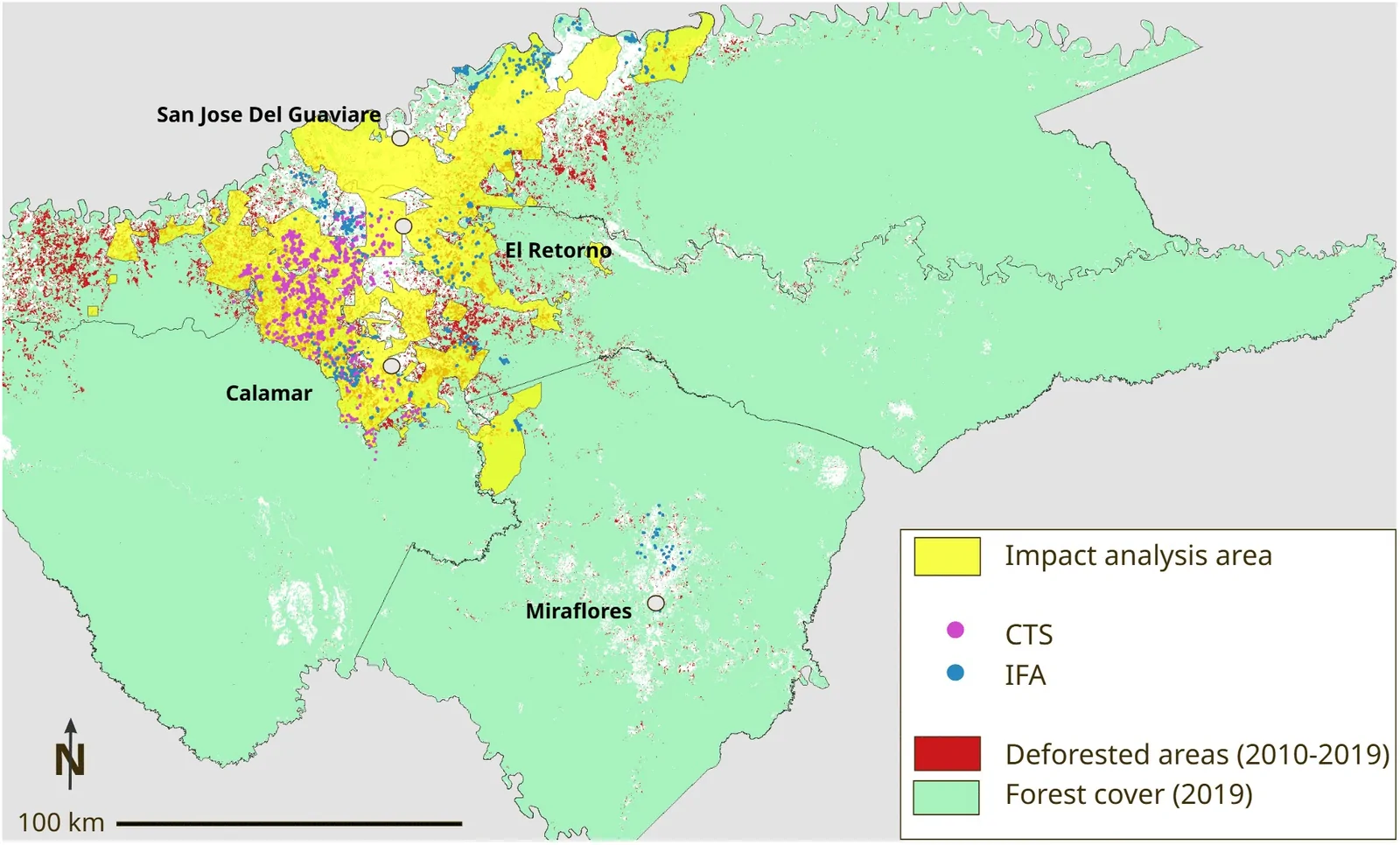
Impact analysis area. Source: authors’ own elaboration using terra and sf packages in R studio, based on data from JRC-TMF and SIAT-AC. Datasets available at https://ies-ows.jrc.ec.europa.eu/iforce/tmf_v1/download.py?type=tile&dataset=DeforestationYear&lat=N10&lon=W80 [Deforestation], https://ies-ows.jrc.ec.europa.eu/iforce/tmf_v1/download.py?type=tile&dataset=UndisturbedDegradedForest&lat=N10&lon=W80 [Forest cover], and https://datos.siatac.co/pages/coberturas [Forest cover in the Colombian Amazon]. Disclaimer: The designations employed and the presentation of material on this map do not imply the expression of any opinion whatsoever on the part of the European Union concerning the legal status of any country, territory, city or area or of its authorities, or concerning the delimitation of its frontiers or boundaries.

SIAT-AC, in contrast, releases land-use and cover maps for deforestation hotspots in the Colombian Amazon every six months, although these maps cover only part of the Guaviare department. SIAT-AC is the only dataset that provides forest-cover information for the areas enrolled in the two programs over the study period (from 2019 to June 2022) and we thus used it to identify our impact analysis area (Fig 6). It classifies areas as forested or deforested, and it does not discriminate between deforestation and forest degradation. As S1 Fig shows, the forest cover levels provided by SIAT-AC sit between the undisturbed forest cover levels and total forest cover levels from the JRC-TMF database (S1 Fig).

To plot the long-term evolution of the deforestation patch size between 1990 and 2021, we created an index using the JRC-TMF database. For each year we computed a deforestation raster that indicates the pixels deforested for the past 12 months. We then measured the mean size of the deforestation patches and divided it by the mean size of the reference period. This index allowed us to observe the evolution of the deforestation patch size and to highlight the difference of sizes before and after the year of the peace accords (2016). However, the index does not reflect the real size of the patch and does not account for the fact that recent deforestation patches are usually an extension of former deforestation patches. To compute the size of the patch during the study period, we used the SIAT-AC database and, drawing on previous deforestation research in the Colombian Amazon [22,41], we created a raster indicating the forested pixels that were deforested during the study period (2018–2022) and computed the mean size of the patch along this period.

To obtain basic information about the IFA and CTS goals and to access key data, we contacted key staff from *Visión Amazonía* and ONF-Andina. They provided us with shapefiles for each participant’s landholding, which also included the total area of protected forest under each contract. For CTS, we additionally obtained shapefiles for some contracts that were signed but never implemented, or that were discontinued shortly after signing because landowners chose not to continue enrolled. We used these cases as a placebo test in our estimations.

Table 3 includes the 10 covariates used for matching purposes and the correspondent data sources. We used the raster of slope and elevation data provided by NASA to compute the distance of each pixel to the closest rivers and protected area, with the latter data obtained from the *Instituto Geográfico Agustín Codazzi* (IGAC). We used data from the government of Colombia to compute the distance to the closest community center and local road, and data from [42] to compute the distance from lights at night. We treated the latter as a proxy of distance to markets and economic activity. We used data from the United Nations Office on Drugs and Crime (UNODC) to obtain the intensity of coca production in each grid cell. Finally, we used the SIAT-AC database to compute past forest cover level and distance to agricultural land.

**Table 3:** Matching covariates and outcome variable, including data sources.

Variable	Source	Unit	Computation	Reference or link to data source
Distance to roads	Instituto Nacional de Vias – Red vial, Government of Colombia	meter	Distance computation	See (1) below
Distance to the center of the main town or a community	Departamento Administrativo Nacional de Estadística (DANE), Government of Colombia	meter	Distance computation	See (2) below
Distance to rivers	Instituto Geográfico Agustín Codazzi (IGAC)	meter	Distance computation	See (3) below
Distance to agricultural lands in 2020	Sistema de Información Ambiental Territorial de la Amazonía Colombiana, Government of Colombia (SIAT-AC)	meter	Distance computation	See (4) below
Coca cultivation	Oficina de las Naciones Unidas contra la Droga y el Delito – UNODC	ha/km²		See (5) below
Natural parks	Parques Nacionales Naturales, Government of Colombia	meter	Distance computation	See (6) below
Elevation	National Space Agency, US government	meter		See (7) below
Slope	National Space Agency, US government	degrees		See (7) below
Distance to lights at night	Li et al., 2020	meter	Distance computation	Ref [42] Dataset. https://doi.org/10.6084/m9.figshare.9828827.v5
Level of forest cover (pre-treatment)	Sistema de Información Ambiental Territorial de la Amazonía Colombiana, Government of Colombia (SIAT-AC)	0:1		See (8) below

1. https://www.colombiaenmapas.gov.co/?e=-86.88181750976501,-4.215092539782901,-61.61326282227172,14.200343161241987,4686&b=igac&u=0&t=39&servicio=103

2. https://www.colombiaenmapas.gov.co/?e=-78.37839954101729,-13.28977155612525,-70.11668079101948,23.03262903632737,4686&b=igac&u=0&t=3005&servicio=690

3. https://www.colombiaenmapas.gov.co/?e=-87.77171008788979,-3.995929952378668,-60.76731555664695,13.98723085234687,4686&b=igac&u=0&t=23&servicio=205

4. https://www.colombiaenmapas.gov.co/?e=-71.94158279837522,1.574014799632258,-71.6585131877799,1.7155770489291435,4686&b=igac&l=3&u=0&t=1&servicio=3

5. https://www.colombiaenmapas.gov.co/?e=-89.32078235351436,-13.289771556125238,-59.218243291022375,23.03262903632737,4686&b=igac&u=0&t=3005&servicio=454

6. https://www.colombiaenmapas.gov.co/?e=-78.37839954101729,-13.376009460790266,-70.11668079101949,22.951042804787186,4686&b=igac&u=0&t=206&servicio=62

7. https://www.earthdata.nasa.gov/topics/land-surface/digital-elevation-terrain-model-dem

8. https://datos.siatac.co/pages/coberturas

### Unit of analysis and outcome variable

We used a quasi-experimental counterfactual-based research design comparing change of forest cover between enrolled sites and similar non-enrolled sites, before and after the beginning of the studied programs. We ran the analysis at grid cell level to overcome the fact that we did not have land-use property data against which to compare our treated landholdings. Grid cells are spatial squares overlapping with participants and non-participants’ landholdings for which we can measure levels of forest cover, deforestation, and other spatially explicit characteristics such as distance to roads, and elevation. Cells are a good proxy to capture land-use decisions at the household level when land-use and property official data do not exist, as is the case for our study region.

We used the same process to define enrolled and non-enrolled cells in the two studied programs, based on the polygons provided by *Visión Amazonía* and ONF-Andina. For each program we defined the treatment variable as a binary variable. Every grid cell with at least 20% of its area registered under one of the programs was considered enrolled. This percentage threshold allowed us to have an important number of observations while not considering grid cells that were almost not treated. For non-enrolled cells the treatment status was always “0”, and for enrolled cells it was “0” until it became “1” when the given cell entered the program. Using a binary variable to define treatment instead of a continuous measure (e.g., the share of the grid cell treated) leads to an underestimation of the effect. Our estimates can thus be considered lower bounds of the two programs’ effects, at least in principle (see limitations below). Furthermore, to make sure that the partial spatial overlap between both initiatives would not jeopardize our identification strategy, we excluded from the analysis grid cells that had more than 40% of their forest area in both programs. Finally, for each cell, we computed the evolution of forest cover using the SIAT-AC database and used such change in forest cover over time as the outcome variable.

### Identification strategy

We applied a generalized DiD design with multiple time periods to estimate the effects of IFA and CTS on deforestation and degradation. A standard two-way fixed effects model is the most common approach:


$$\begin{array}{c} Y_{it}=\theta_{t}+\eta_{i}+\alpha_{p}T_{pit}+\varepsilon_{it} \end{array}$$

where $Y_{it}$ is the level of forest cover for individual iat time t, $\theta_{t}$ denotes time fixed effects, $\eta_{i}$ individual fixed effects, and $T_{pit}$ the treatment status of individual i in program p. In this framework, $\alpha_{p}$ identifies the average effect of program p.

However, this basic specification does not guarantee consistent estimates for two reasons. First, because enrolment in IFA and CTS is (or was) voluntary, participants may differ systematically from non-participants. For example, those who choose to engage in conservation are likely to be landholders with lower opportunity costs of avoiding deforestation—that is, they would have cleared less forest even in the absence of the initiatives. Directly comparing treated and untreated areas would therefore overestimate the level of avoided deforestation. Second, the effect of the initiatives on avoiding deforestation are likely to be highly heterogeneous, reflecting complex socio-ecological dynamics. In such cases, the classical DiD estimator is inconsistent in settings with staggered adoption because it implicitly averages heterogeneous effects with potentially negative weights [43].

To address these potential biases, we used propensity score reweighting [44], which constructs a control group with the same treatment probability as the treated group, conditional on observables. To increase robustness, we applied a doubly robust estimator, which combines reweighting with regression adjustment [45,46]. This ensures validity if either the weighting or regression specification is correctly specified. We also applied the Callaway and Sant’Anna (CSA) estimator (ibid.), which allows for multiple cohorts, multiple periods, and heterogeneous treatment effects in a staggered DiD model. Such estimator has several advantages because it relies on the CPTA, as well as it accommodates treatment anticipation by allowing the reference period to precede the actual treatment start, and it decomposes the ATT into group–time-specific effects.

As a result, the group–time ATT is defined as:


$$\begin{array}{c} ATT_{p}(g,t)=E[Y_{t}(\text{1})-Y_{t}(\text{0})|G=g,T=t] \end{array}$$

where G indexes treatment cohorts. For example, ATT_ONF (2019.5, 2020.5) denotes the effect of CTS on the June 2019 cohort one year later. These group–time ATTs can then be aggregated into cohort averages, time averages, or event-study estimates. In the case of IFA, and since all contracts began in June 2021 (2021.5), we only considered a single cohort, with our parameter of interest being:


$$\begin{array}{c} ATT_{IFA}=\;ATT_{IFA}(\text{2}\text{0}\text{2}\text{1}.\text{5},\;\text{2}\text{0}\text{2}\text{2}.\text{5}) \end{array}$$

which, in this case, is equivalent to a standard doubly robust DiD estimate [45].

In the case of CTS, three cohorts entered in mid-2018, early 2019, and mid-2019, with the program ending in June 2022. We estimated the cohort-specific effects in 2022 and aggregated them as a year-average ATT:


$$\begin{array}{c} ATT_{ONF}=\;\theta_{ONF}^{year}(\text{2}\text{0}\text{2}\text{2}.\text{5})=\;\sum_{g}w_{g}ATT_{ONF}(g,\text{2}\text{0}\text{2}\text{2}.\text{5}) \end{array}$$

with $w_{g}=\;\frac{N_{g}}{N}$ the share of each cohort.

Finally, to compare the two programs’ effect on an annualized base, we computed the event study average treatment effect for each group: $\theta_{ONF}^{ES}(\text{3})$ and $\theta_{IFA}^{ES}(\text{1})$ such that:


$$\begin{array}{c} \theta_{ONF}^{ES}(\text{3})=\;\sum_{g}w_{g}ATT_{ONF}(g,\;g+\text{3}) \end{array}$$

$$\begin{array}{c} \theta_{IFA}^{ES}(\text{1})=\;ATT_{IFA} \end{array}$$

We then annualized those effects:


$$\begin{array}{c} ATT_{ONF}^{annual\;}=\;{\left(\frac{\text{1}+\theta_{ONF}^{ES}(\text{3})}{\text{1}}\right)}^{\frac{\text{1}}{\text{3}}} \end{array}$$

$$\begin{array}{c} ATT_{IFA}^{annual\;}=\;\theta_{IFA}^{ES}(\text{1})=\;ATT_{IFA} \end{array}$$

### Standard errors, robustness checks and placebo test

We used simultaneous confidence bands and to compute them we used critical values which were higher than for classical confidence intervals, as recommended in [46]. In our case the critical value for a 95% uniform confidence band was 2.73 instead of 1.96. Moreover, we clustered the standard errors at the *vereda* level to account for spatial autocorrelation.

We tested 162 additional models to verify the robustness of our results to alternative model specifications, and we also examined two alternative grid-cell sizes (5 ha and 25 ha). Because our main specification included all pixels with at least 20% forest cover at the start of the period, we also tested thresholds of 10% and 40%. We further assessed two alternative cut-offs for defining treatment status (20% and 60%), compared with the original 40%. In addition, we estimated models that did not allow for anticipation effects. Finally, we tested different levels of overlap between programs. In the main specification, we excluded any treated cell with more than 40% of its forested area enrolled in a program other than the one being evaluated. As robustness checks, we assessed two stricter restrictions: excluding cells with more than 20% overlap with the other program and excluding any overlapping cells entirely.

To assess the plausibility of the CPTA, we implemented the sensitivity analysis proposed by [47], imposing restrictions on the magnitude of allowable post-treatment deviations in trends between treated and control units. This “honest DiD” framework quantifies the extent to which violations of the CPTA can be accommodated without overturning statistical inference (ibid.). In our setting, a key concern is that deforestation trends may differ systematically across groups. Treated units may have a lower underlying deforestation risk and thus exhibit more moderate trends even in the absence of treatment, leading to differential dynamics that are not attributable to the intervention.

Let’s denote ∆(M) the interval of possible values of the treatment effect at the 95% threshold given M, M being the amount by which the slope of differential trend can vary. To do so we used the $\Delta^{SD}$ estimation [46]. Our parameter of interest is therefore:


$$\begin{array}{c} {\overset{―}{M}}_{p}=\;\underset{\text{0}\;\Delta (M)}{\text{max}}\left(M\right) \end{array}$$

With p the program of interest, the maximum deviation accepted so that 0 is not in Δ(M); in other words, so that the effect we measure is still significant at the 5% threshold.

We also had access to data on 43 land plots that were characterized by CTS but never enrolled in the program or exited at an early stage. We used these plots as a placebo test, as they were expected to be very similar to the treated parcels but did not receive payments. If we were to find a significant treatment effect in these areas, this would suggest that our estimated impact is driven by unobservable factors rather than the treatment itself. We applied the same estimator as for CTS; however, in this case, a grid cell was considered treated if it was characterized but not enrolled.


$$\begin{array}{c} ATT_{Placebo\;ONF}=\;\theta_{Placebo\;ONF}^{year}(\text{2}\text{0}\text{2}\text{2}.\text{5})=\;\sum_{g}w_{g}ATT_{Placebo\;ONF}(g,\text{2}\text{0}\text{2}\text{2}.\text{5}) \end{array}$$

Finding a significantly positive estimate of *ATT*_*Placebo ONF*_ would indicate that our estimation strategy identified an effect for a placebo treatment—i.e., that our estimation strategy led to a significant upward bias.

### Software

We obtained estimates and graphs using the R package did, version 2.1.1 (https://bcallaway11.github.io/did/index.html). Robust analytical standard errors were clustered at the *vereda* level in each specification. For our robustness checks, we used the spec_chart R package (https://github.com/ArielOrtizBobea/spec_chart) to obtain specification charts. Finally, we used the honestdid R package (https://github.com/asheshrambachan/HonestDiD) and the wrap-up created by Pedro Sant’ Anna (https://github.com/pedrohcgs/CS_RR) to check the robustness of the CPTA.

### Internal and external validity

The assumption least likely to hold in our analysis is the CPTA, as our list of matching covariates was not exhaustive. We were unable to map and control for illegal roads, which may have led to an underestimation of the treatment effect. Deforestation risk is often higher in properties closer to illegal roads and the probability to enroll in the program may also be higher along such roads because of increased accessibility and access to policy programs and projects. We were also unable to match on households’ socio-economic characteristics, as such data were unavailable for non-enrolled households and parcels in our study region.

Because of such data availability restrictions, we ran the analysis only for a sub-region of Guaviare where deforestation risk is expected to be higher. Therefore, our results for the IFA program may not be valid for the entirety of Guaviare. In this case, the overall effect might be lower than the effect measured for the impact analysis region, since some of the areas falling outside the impact analysis area are rather remote and deforestation risk may be lower.

## Results

### Compliance and impact

**Fig 7:**
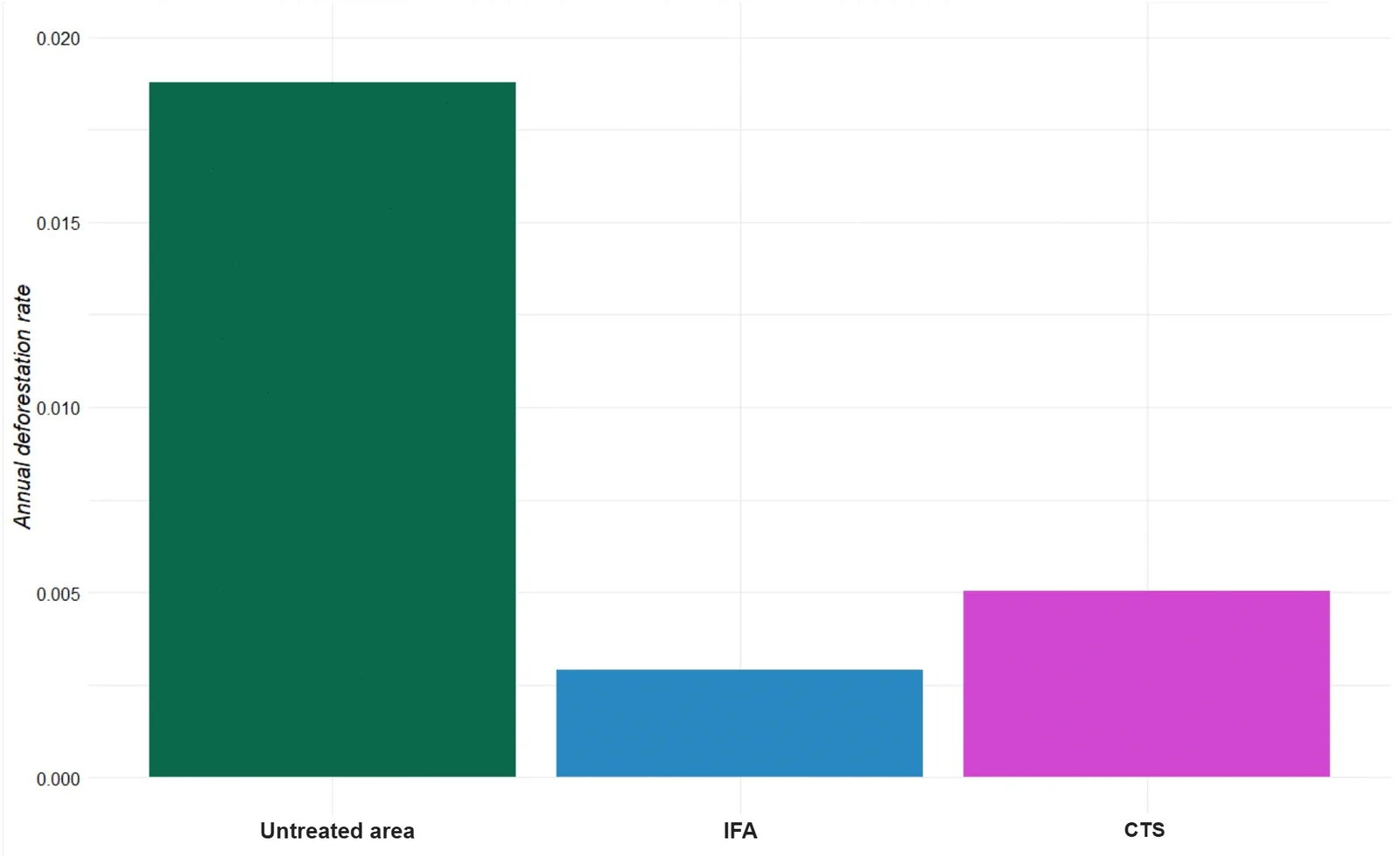
Compliance of IFA and CTS over the study periods. Caption: The graph displays the annualized deforestation rates in conservation areas of enrolled parcels for each program. The untreated area represents forests that are outside treated parcels.

We observe high levels of compliance in both initiatives during the treatment periods: 2021–2022, for IFA; and 2019–2022, for CTS (Fig 7). In the areas not treated by either program the annual deforestation rate is around 1.8%, but it is at least three times lower in areas enrolled in IFA and CTS. Specifically, the average annual deforestation rate in IFA’s enrolled areas is 0.28% versus 0.5% for CTS enrolled areas. This suggests that the degree of compliance is slightly higher for IFA than CTS.

**Fig 8:**
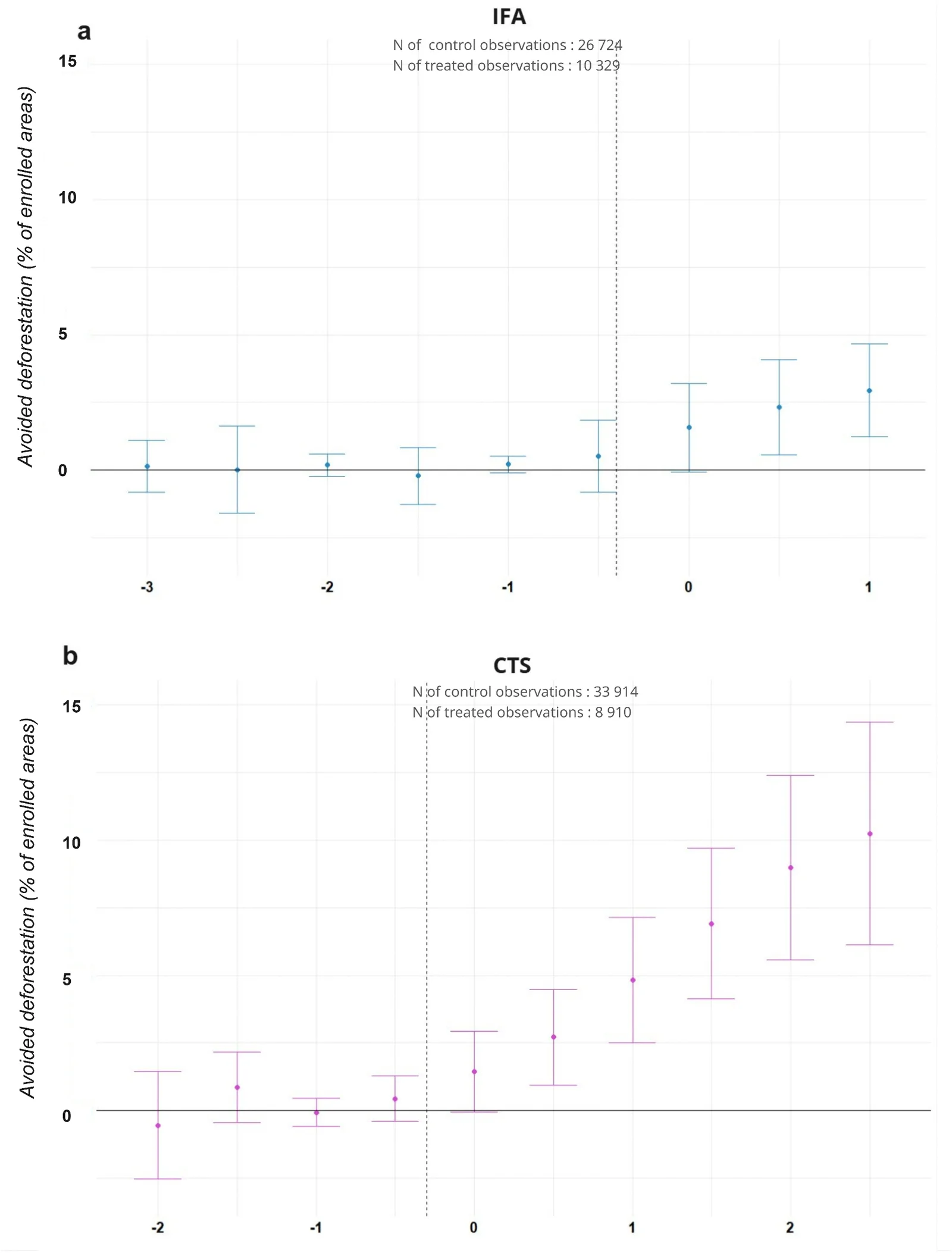
Impact of the IFA and CTS programs on avoided deforestation. Caption: The graph displays the event study impact of the programs. The dotted line represents the beginning of the program and t = 0 marks the beginning of the contracts analyzed, 2021-2022 for IFA and 2018/2019-2021/2022 for CTS. The impact on avoided deforestation of IFA on enrolled parcels is observed one year after implementation started and slightly increases over the evaluation period. The impact of CTS also starts straight after implementation and increases over the 3-year impact analysis period.

On average, forest cover is 2.9% higher in areas enrolled in the IFA program compared to non-enrolled, control areas facing similar deforestation pressures (95% confidence interval [1.51:4.38]) (Fig 8a). We therefore estimate that approximately 276 ha of forest cover were preserved by the program between 2021 and 2022. The magnitude of the effect is four times greater in the CTS project area, where forest cover is 12% higher in enrolled areas compared to non-enrolled control areas (CI: [8.33: 15.6]) (Fig 8b). This represents approximately 1,130 ha of avoided deforestation in the CTS targeted area between 2019 and 2022.

**Fig 9:**
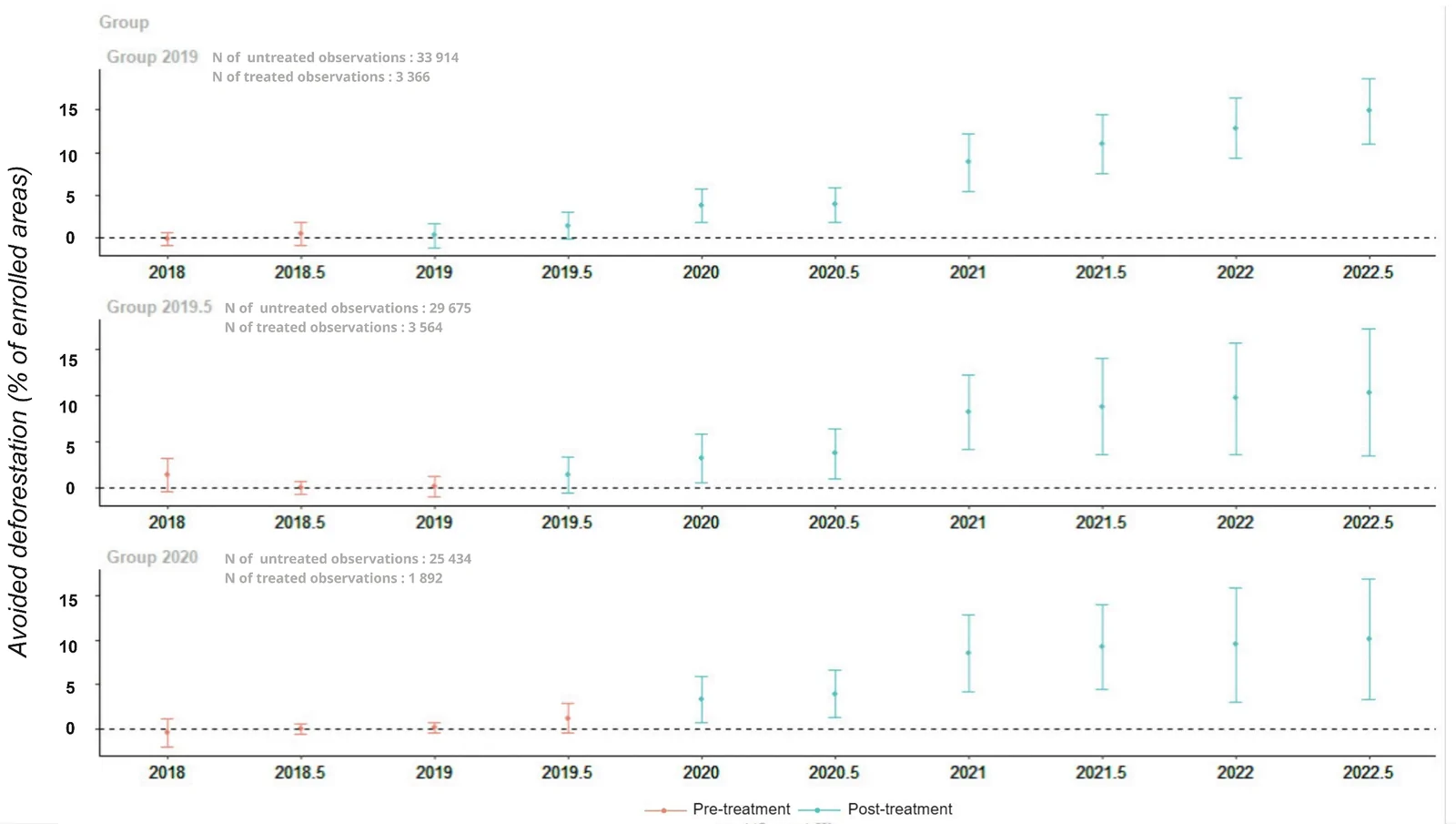
Impact of the CTS program disaggregated by participant cohort expressed in percentage of enrolled areas.

Fig 9 further presents the disaggregated impact for each cohort of the CTS initiative. For all cohorts, the treatment effect at the end of the program (2022) exceeds 10% and is statistically different from zero. The effect increases over time: the estimated effect for enrolled areas in the 2019 cohort is approximately 14.8% points (CI: [11.1, 18.7]), whereas it is 10.1% points for enrolled areas in the 2020 cohort (CI: [3.06, 17.1]).

The difference in impact across the two studied initiatives can be explained by the length of the study periods (one year for IFA versus three years for CTS), as well as by their annualized effects: 2.9% of enrolled areas for IFA and 3.8% of enrolled areas for CTS. Additionally, we can extrapolate these effects to the entire department using the estimated ATT, as noted earlier. Consequently, the difference in total impact becomes smaller in terms of avoided deforestation, as the number of contracts included in the analysis differs between the initiatives: 90% of the CTS contracts fall within our impact analysis area, while only 58% of the IFA contracts do so. Table 4 summarizes these results.

**Table 4:** The IFA and CTS programs’ effectiveness compared.

	IFA	CTS
ATT (% of enrolled areas) *95% Confidence interval*	2.90 [1.51: 4.38]	12.0 [8.33: 15.6]
Annualized ATT (% of enrolled areas) *95% Confidence interval*	2.90 [1.51: 4.38]	3.80 [2.70: 4.95]
Total avoided deforestation in the impact analysis area (ha) *95% Confidence interval*	276 [142: 412]	1,130 [784: 1470]
Avoided deforestation in Guaviare (extrapolated ha)	499	1440
Number of observations	37,053	42,824
Number of treated observations	10,329	8,910
Number of cohorts	1	3

### Sensitivity and robustness analyses

We assess the robustness of the treatment effect to a relaxation of the CPTA using the “honest” DiD approach [47] (Table 5). For the CTS, a period-to-period difference in deforestation trends of 0.04 between treated and control groups does not compromise the statistical significance of our results. This implies that, even if deforestation rates in the treated group were up to 80% lower than in the control group in the absence of treatment, the estimated effect would remain significant. However, we do not find the same level of robustness for the IFA initiative. In this case, deforestation rates in the treated group would need to be only 8% lower than in the control group, in the absence of treatment, for the effect to remain significant.

**Table 5:** Honest DiD results for the IFA and CTS programs.

	IFA	CTS
${\underset{―}{M}}_{g}$	0.002	0.04
Maximum tolerated deviation in terms of deforestation rates	8%	80%
Number of observations	37,053	42,824
Number of cohorts	3	1

Caption: We translated the results of the “honest” DiD in terms of forest cover change between treated and untreated cells. An 80% maximum tolerated deviation means that the annual rates of deforestation between treated and untreated areas could differ by up to 80% without jeopardizing the significance of our results.

**Fig 10:**
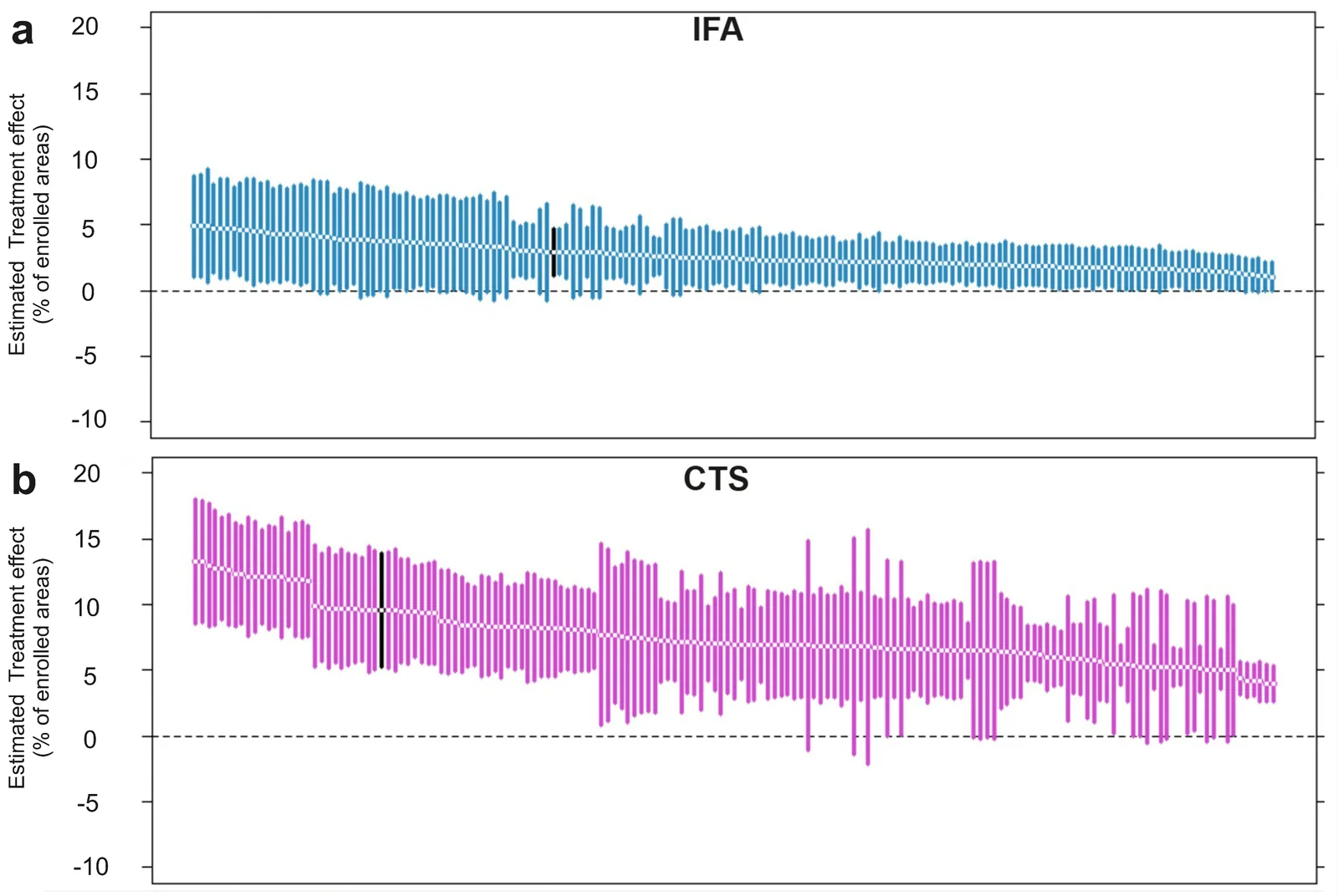
Testing alternative model specifications for the IFA and CTS programs. Caption: We plot the event study estimates obtained on the 163 programs tested with the 95% CI band for both initiatives. Our main specification estimation is highlighted in black. The estimates are ranked in a decreasing order. See S2 Fig and S3 Fig for details on each specification.

Subsequently, to ensure our results are not determined by a specific model choice, we run the estimations for 162 alternative models that could also be valid a priori. For IFA, we find that 88% of all the tested models lead to positive and significant estimates. The mean estimated effect is 0.027, which is close to our main estimate: 0.029 (Fig 10a). For CTS, all the tested specifications lead to an ATT that is significant at the 95% threshold. Out of the 193 specifications, the mean value of the ATT is 0.076, against 0.12 with our main specification. The model we chose as main specification leads to higher values for the ATT than 80 of the alternative ones (Fig 10b).

These findings suggest that the significance of the treatment effect is stable to changes in specification for both initiatives. However, in terms of magnitude, the effect of the CTS is significantly higher in our main specification, indicating that there may be considerable uncertainty around the true value of the program’s average impact. We therefore cannot exclude the possibility that our main estimate overstates its effect on avoided deforestation.

**Fig 11:**
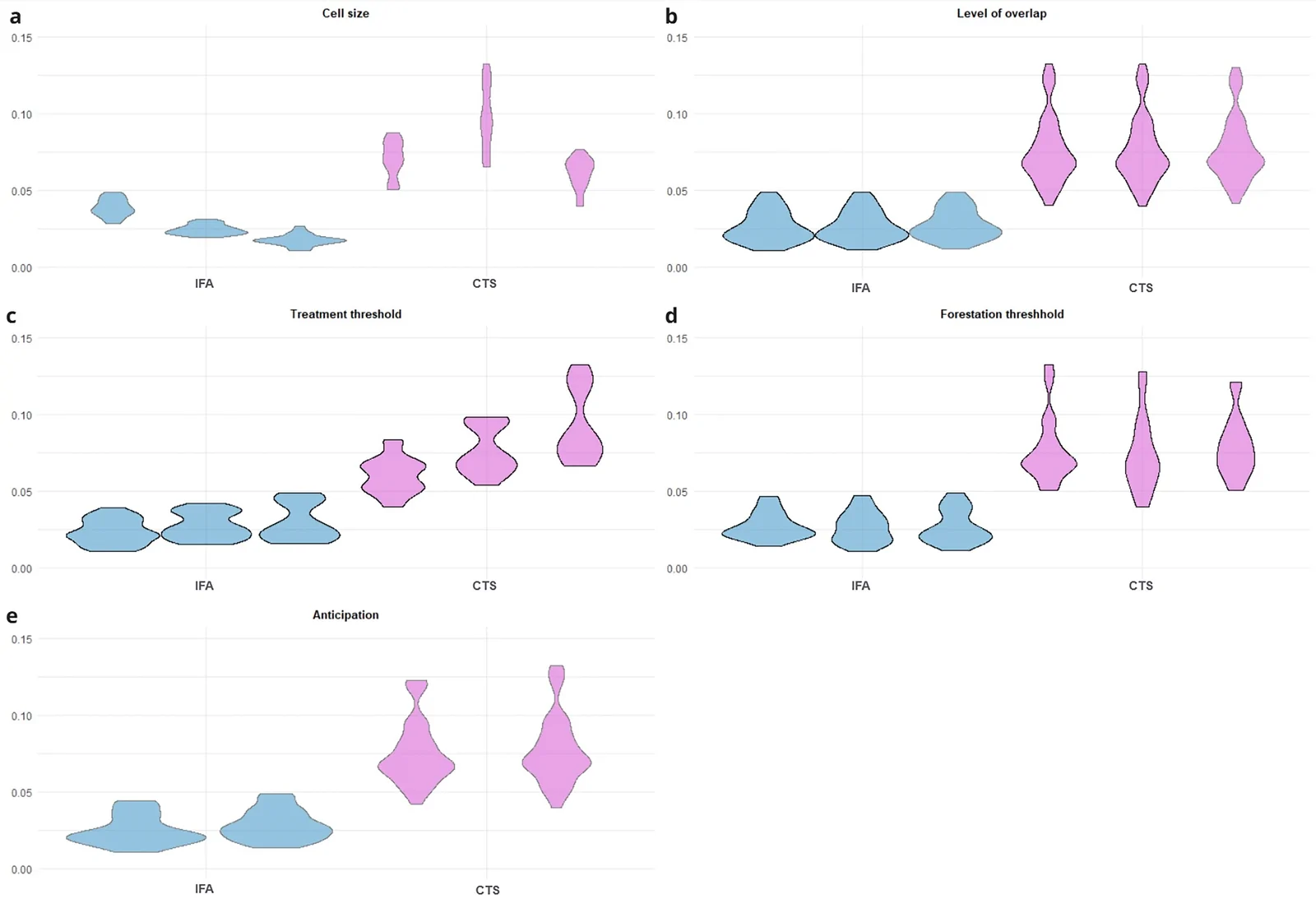
Determinants of the variability of estimates across model specifications. Caption: The alternative estimates are obtained by varying five different parameters. In each panel of the figure, we plot, for both programs, the distribution of estimated effects across specifications. Panel A displays, from left to right, the distribution of estimates using 5 ha, 10 ha, and 25 ha grid cells. Panel B presents results across different levels of overlap. Panels C, D, and E show, respectively, the sensitivity of the estimates to the treatment definition threshold, the forest cover threshold, and the inclusion of anticipation effects.

To further investigate this issue, we examine how each parameter change affects the estimates. Fig. 11 shows that the parameters with the greatest influence on the estimates are the definition of the treatment variable and the size of the grid cell. For both initiatives, the higher the threshold used to define treatment (which we set at 40%), the higher the estimated effect. This sensitivity to the treatment definition can be explained by the fact that, when considering only the pixels with most of their area enrolled in the initiative, we focus on the cells that are most intensely treated, i.e., like a dose-response relationship. The results are less intuitive for the size of the grid cell. Although grid-cell size is the parameter that explains most of the variation across specifications for both initiatives, its effect on the magnitude of the estimate diverges between them. For IFA, the estimate decreases as pixel size increases, and the highest ATT estimates are obtained with a 5 hectares cell size. In contrast, for CTS, the highest values are produced using 10 hectares cells (the size used in our main specification). This pattern may reflect differences in parcel sizes across the two initiatives: parcels enrolled in CTS tend to be larger than those in IFA (69.5 ha *versus* 50.5 ha on average). However, these results remain difficult to interpret because the relationship between coefficient magnitude and scale also depends on the correlation between covariates and treatment [48].

### Placebo test for the CTS program

**Fig 12:**
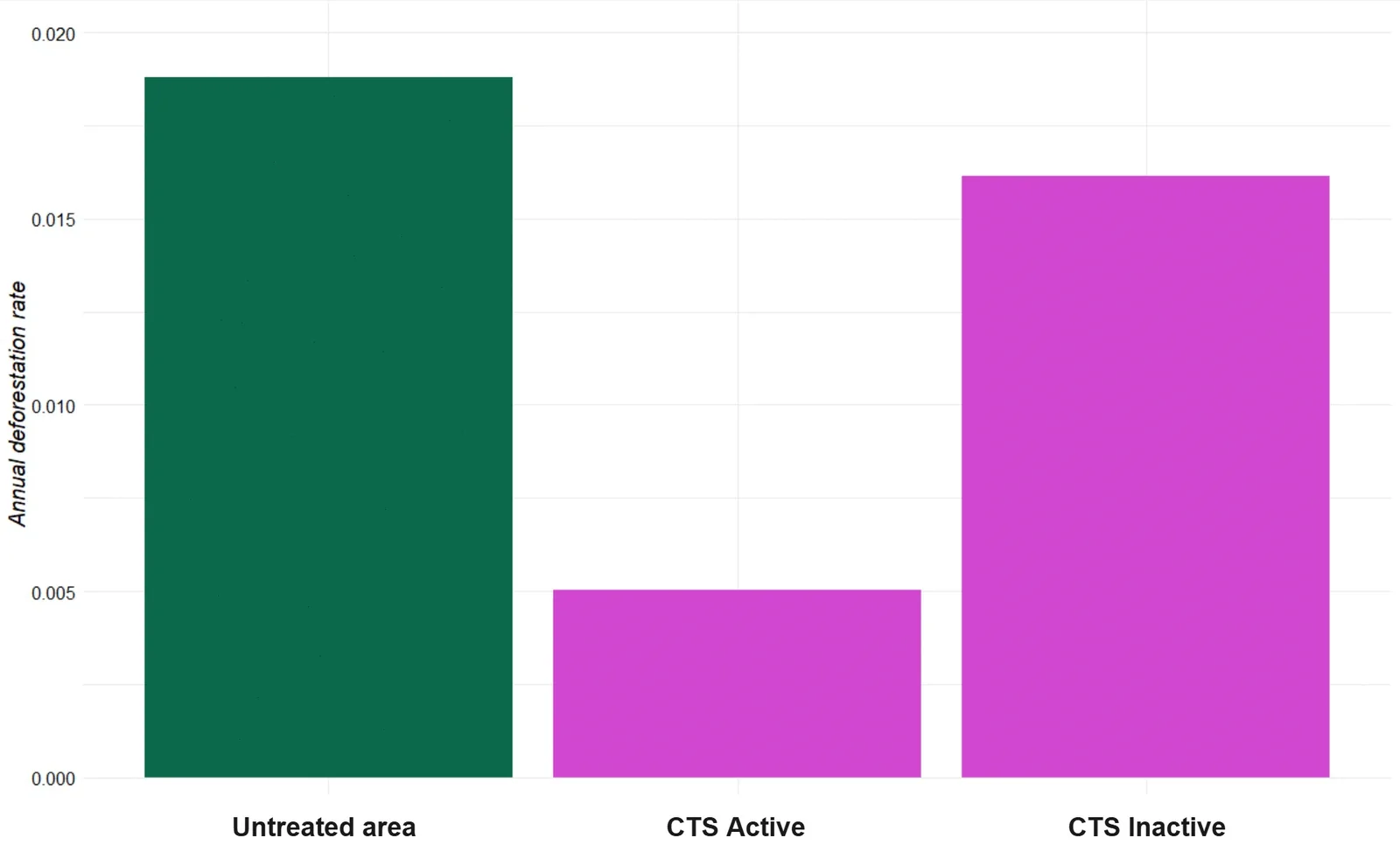
Deforestation rates for the CTS placebo test.

By running the placebo test with CTS parcels that ultimately did not enroll, we observe that the effect of being in an untreated cell is close to zero and not statistically significant (Table 6 and Fig 12). However, the low number of observations for the placebo test (429 compared with 8,910 for the main CTS specification) limits the precision of these estimates. We also observe that the placebo CTS parcels exhibit deforestation rates like untreated areas and higher than those of parcels enrolled in CTS, which suggests that remaining in the program influences compliance.

**Table 6:** Placebo test results for the CTS program.

	CTS active	CTS inactive (placebo)
ATT *95% Confidence interval*	12.0 [8.33: 15.6]	1.89 [-16.9: 20.8]
Annualized ATT *95% Confidence interval*	3.80 [2.70: 4.95]	0.626 [-5.34: 6.50]
Number of observations	42,085	42,824
Number of treated observations	8,910	429
Number of cohorts	3	3

## Discussion and conclusion

### Summary of findings

Our findings add to existing evidence on the effectiveness of PES programs for forest conservation [6,13,16]. We have shown that two distinct PES initiatives aimed at avoiding deforestation in the Colombian Amazon can be modestly successful. The initiatives studied have saved approximately 1,406 ha of forest in the study area over a 2–3-year period. This positive though very modest impact, however, seems minimal when compared with the number of hectares deforested in Guaviare recently, averaging approximately 5,000 hectares per quarter over the past five years. We have also shown that the NGO-run CTS project, during its short lifespan, had a greater impact on avoiding deforestation than the government-run IFA program, with the former’s annualized effect being about four times higher. It would be misleading, of course, to conclude that CTS has outperformed IFA, as the two initiatives differ in the type of incentives provided, their duration, geographical focus, and overarching goals. All these together may contribute to explain the difference in the level of compliance and impact of the two initiatives, to a different, albeit unknown, degree.

IFA is a PES scheme implemented within the broader policy framework of Colombia’s REDD+ strategy. In contrast to other PES programs, however, IFA’s payment levels are related to the proportion of forest allocated for conservation within a participant’s landholding, rather than to the total number of hectares enrolled. The CTS initiative, by contrast, offered technical assistance for the development of land-use plans and it co-financed the conservation, restoration and silvopastoral activities included in such plans. As a result, CTS targeted landowners engaged in productive farming, where the likelihood to convert remaining forest areas into cattle grazing or crop cultivation areas may be higher than in the more remote areas targeted by IFA. The IFA program did not require such a plan, meaning that individuals without productive activities -and therefore with a lower likelihood of shifting land-use- were also likely to participate. This is supported by evidence showing that CTS participants had substantially less forest cover on their properties than IFA participants. Furthermore, the higher compliance levels observed in IFA can be attributed to the lower baseline deforestation risk in the program’s enrolled parcels.

The duration of treatment may also help explain the observed differences in effect. The CTS was a short-term PES project characterized by a high level of field presence by ONF-Andina staff, and the annual revenue received by each participant was also higher than that of IFA. This may have prompted stronger forest conservation efforts on participants’ farms which, combined with a higher baseline risk of deforestation, may have resulted in a greater annualized impact. However, we acknowledge that our analysis of IFA’s impact is limited to a single cohort.

The programs’ distinct targeting strategies may also contribute to explain the higher annualized impact on avoiding deforestation of the CTS project. As noted earlier, the latter was implemented in a relatively small area in northwestern Guaviare, at the intersection of three municipalities. Forests here are already quite fragmented, and deforestation rates have historically been high. The IFA program, in contrast, operates across the entire department, including areas with relatively low human presence, high forest cover, and minimal fragmentation. These forests are also located farther from roads and towns.

Finally, the initiatives’ contrasting goals may also contribute to explain their uneven conservation performance. The CTS project primarily supported farmers in transitioning from unsustainable to more sustainable production methods, whereas the IFA program was conceived as a monetary recognition for environmental stewardship in the socially and politically contested Amazon region. By mid-2023, IFA had already engaged more than 10,000 families across the five targeted Amazonian departments and aims to involve as many of these as possible in the development of forestry enterprises, known as *núcleos de desarrollo forestal y de biodiversidad* (forest and biodiversity development clusters). This broader, long-term ambition explains why IFA targeted all municipalities and most *veredas* of the Amazon region, and why it prioritized more isolated areas away from the agricultural frontier. IFA is a policy program where returns on ecological effectiveness cannot be disentangled from other socio-political objectives, such as equity or the mitigation of social disputes in the context of intermittent armed conflict [17].

Taken together, these findings suggest that IFA and CTS have both had a positive but modest conservation impact, yet the limitations in the number of matching covariates employed and the spatial reach of our impact analysis area may have led to an overestimation of their annualized effect on avoided deforestation. It is also important to stress that their distinct design and implementation features, including different targeting and baseline deforestation risk, type of incentive (only cash vs. cash, technical assistance and land-use planning), exposure length and timing of cohorts, as well as broader objectives, would make such comparison futile. Instead, we argue that the two studied programs have reinforced one another in a region characterized by a weak, often conflict-ridden, land governance context [32]. Future research should investigate whether the IFA annual effect has increased or decreased over time as more participants joined or abandoned the program and, concurrently, compare these effects with the levels of permanence observed in previously treated CTS areas.

### Policy implications

PES programs and projects are not a panacea for forest conservation in the Colombian Amazon, nor beyond [49,50]. The hectares of forests conserved by IFA and CTS in our study area represent an insignificant fraction of the total forest loss in Guaviare during the same period (90,561 ha in total). Government and NGO efforts to curb deforestation struggle to compete with, or confront, the underlying drivers and incentives shaping regional land-use change dynamics. Based on our estimates, deforestation in Guaviare between 2018 and 2022 resulted from land conversion in patches of 20 hectares or larger, which can only be undertaken with considerable capital, labor, and political connections to access or grab land. This is consistent with government figures suggesting that around 50% of deforestation is linked to land conversion for cattle grazing and land speculation, mostly driven by distant, non-local and, in some cases, illegal actors [51].

Curbing deforestation in the Amazon therefore requires more than well-intentioned and effective policies and projects. In our view at least three urgent actions are needed. First, the government’s land-use regulatory agencies should tackle the poor levels of governance and weak enforcement of relevant forest conservation legislation. “New” armed groups are controlling vast areas of the Amazon region and preventing people from participating in state-led environmental and social development programs. Some analysts have even suggested that the historically low deforestation rates observed in 2022 and 2023 were due, in part, to coercion by this group as part of a strategic maneuver in the context of a new wave of peace negotiations [52–54]. While advances in remote sensing have opened new opportunities for near-real-time detection and sanctioning of deforestation [55,56], they risk becoming mere technological showcases with limited impact on the ground, particularly if such advances are not paired with prompt and socially just enforcement.

Second, governments at national and departmental levels should collaborate more with local communities, ensuring that their rights and knowledge are at the center of conservation efforts. Some progress has been made in this regard, as exemplified by the recently launched *Conservar Paga* initiative in the Colombian Amazon -an extension of the IFA program-. Despite its rights-based approach and the Colombian government’s intention to provide some form of basic income for nature conservation [57], the environmental additionality, permanence and spillover effects of this new initiative remain to be seen. This effort should also be paired with structural economic and legal reforms, including comprehensive land registries and credit systems that could discourage large-scale cattle ranching activities and other extractive practices, including illicit ones [58].

Third, it is critical to better integrate rural development and environmental policies. The number of livestock heads in Guaviare increased by 95% between 2016 and 2023, reaching a current total of approximately 500,000 cattle [59]. During the same period, agricultural credit allocated to support cattle ranching activities increased fourfold between 2015 and 2023 [60]. Such increased lending practices may also be behind our observed progressive increase in the size of deforestation patches in the department.

To conclude, we have provided novel evidence on the effectiveness of two PES initiatives aimed at curbing deforestation in Colombia’s department of Guaviare. We have shown the long-term conservation potential of a government-led program but also hinted at the missed opportunity of realizing larger conservation benefits through an NGO project because of a too-short implementation period. Therefore, we encourage the continued implementation of the IFA program as a long-term, government-led anti-deforestation strategy and we also advocate for increased and sustained support for NGO initiatives such as CTS, which can be effective in achieving conservation goals while supporting rural communities’ land-use plans and livelihoods. Beyond the studied region of Guaviare, our comparative analysis provides grounded insights that inform broader debates on the design of large-scale, multi-actor forest conservation and climate mitigation strategies. The differences observed between IFA and CTS -particularly in their targeting, incentive structures, governance arrangements, and implementation timeframes- show that conservation outcomes are shaped not only by the presence of incentives, but by how these are specifically designed and delivered across institutional levels and local contexts.

We have highlighted the value of adopting a ‘mix-and-match’ approach where PES initiatives are combined across scales and actors to address heterogeneous drivers, motivations, and opportunity costs. Such intervention complementarities are also consistent with jurisdictional and nested REDD+ approaches, which emphasize the alignment of policies and incentives across governance levels while allowing for locally adapted implementation [61]. From a financing perspective, our findings highlight that the effectiveness of large-scale forest conservation instruments depends not only on the amount of funding mobilized as a necessary condition, but most importantly on how incentives are targeted, the types of support provided, the governance arrangements though which programs are implemented, and the duration of funding commitments. These design features are central to emerging large-scale initiatives that seek to mobilize substantial funding for forest conservation -like the Tropical Forest Forever Facility- but which must ultimately make critical decisions regarding incentive design, monitoring and evaluation mechanisms, and the articulation of multiple implementing actors and instruments. Our results suggest that careful attention to how financing mechanisms are designed, targeted, sequenced, and implemented across governance levels and local contexts will be essential for achieving both effective and enduring conservation outcomes in tropical forests.

## Supporting information

S1 Fig JRC and SIAT-AC [SINCHI] data compared. (TIF)

S2 Fig Model specifications for the IFA program. (TIF)

S3 Fig Model specifications for the CTS program. (TIF)
