# Correction: Incentives are effective but insufficient to halt smallholder deforestation in the Colombian Amazon

**DOI:** 10.1371/journal.pone.0362566

**Published:** 2026-11-10

**Authors:** Hugo Charoud, Esteve Corbera, Lina Moros, Sébastien Costedoat, Santiago Izquierdo-Tort

In Table 5, there is a mistake in row 5. Number of cohorts should indicate 1 for IFA and 3 for CTS. Please see the correct Table 5 here.

**Table 5:** Honest DiD results for the IFA and CTS programs.

	IFA	CTS
${\underset{―}{M}}_{g}$	0.002	0.04
Maximum tolerated deviation in terms of deforestation rates	8%	80%
Number of observations	37,053	42,824
Number of cohorts	1	3

Caption: We translated the results of the “honest” DiD in terms of forest cover change between treated and untreated cells. An 80% maximum tolerated deviation means that the annual rates of deforestation between treated and untreated areas could differ by up to 80% without jeopardizing t 0340539 he significance of our results.
