# Supplementary material for: Incentives are effective but insufficient to halt smallholder deforestation in the Colombian Amazon
Source: PLoS One. 2026 Sep 23;21(9):e0340539. doi: 10.1371/journal.pone.0340539 (PMC13600516; doi:10.1371/journal.pone.0340539)
Supplement: Supplementary file 1 [file pone.0340539.s004.pdf]

Manuscript: Incentives are effective but insufficient to halt smallholder deforestation in the Colombian Amazon

Overall Assessment

This manuscript evaluates the impact of two initiatives aimed at deterring deforestation in the department of Guaviare, Colombia: the government-led Incentivo Forestal Amazónico (IFA) and the NGO-led Caminemos–Territorios Sostenibles (CTS).

The manuscript presents an original, spatially explicit impact evaluation of two overlapping but independently implemented initiatives in the Colombian Amazon, using recent advances in difference-in-differences and sensitivity analysis. The manuscript is methodologically strong and makes a valuable contribution to the literature on the effectiveness of PES.

General

1. The data availability statement indicates that some data are held by third parties. It would be helpful to provide links or additional information for these sources.
2. The ethics statement is listed as N/A. However, the authors report "*interviews with staff from IFA and CTS to gain access to key documentation and obtain information about program goals, eligibility rules, and implementation practices*", which needs clarification regarding an IRB (Institutional Review Board) and/or exemption.

Introduction

3. Explicitly state research questions/objectives of the study. A summary table linking research questions, estimates, data sources, and figures could improve accessibility.
4. Please clarify the connection between:

The conceptual logic from PES theory => expected mechanisms => testable expectations

This clarification will help explain why comparing cash transfers (IFA) with technical assistance/land-use planning (CTS) is theoretically meaningful rather than only empirically convenient.

5. The introduction summarizes PES generally and then jumps to "mixed evidence." Consider adding 2–3 sentences that explain mechanisms by which incentives should reduce deforestation (increasing relative returns to conservation, adoption of lower-deforestation practices, changing land-use planning/monitoring), and why those may fail (high opportunity costs, weak enforcement/tenure, powerful external actors).
6. It is not clear why the two initiatives under analysis are labeled "PES". CTS is described as technical assistance and land-use plans (in-kind payments). This classification may fit "incentive-based conservation" broadly, but it might not be considered PES in the strict conditional-payment sense. Please justify the classification (conditional incentives linked to conservation commitments/land-use plans) or adjust the language to "incentive-based conservation programs".
7. Consider elaborating on the research gap. As it is, the introduction only states that three studies analyze PES effectiveness in Colombia's Amazon and none examines overlapping initiatives. It is a useful and informative gap, but it would be stronger if you specified what is unknown:
    (a) Is the gap mainly due to differences in comparative effectiveness between different incentive types (cash vs in-kind/TA)?
    (b) Or policy-mix interactions/overlap (multiple programs in the same landscape)?
8. I suggest avoiding broad quantitative statements without context/citations. For example, "…can globally release as much CO_2 annually as all EU countries

combined". This sentence may be directionally right, but it needs to be contextualized (year? includes degradation? includes soil or other sectors?). Consider including a more standard statement on global emissions from deforestation/land-use change (with an updated citation if needed).

9. The introduction would benefit from a short paragraph situating IFA within the historical evolution of Colombia's national REDD+ framework, including the timeframe, to help explain the policy logic behind the program's design and targeting.

Material and Methods

10. Fix typos: enrolment, enrol.
11. I suggest defining acronyms once and consistently: CPTA/CPT, DR, CSA, etc. are used heavily. Please add a short "Notation/abbreviations" sentence or ensure consistent naming ("conditional parallel trends assumption" vs "CPTA").
12. The context section is strong, and the historical narrative is informative. However, some details (rubber company history, colonization waves) may be more appropriate for the Introduction/Background or Discussion, unless they are directly linked to the identification or selection of covariates.
13. Please clarify the "treatment" concept. IFA appears closer to classic conditional payments, and CTS is a bundle of planning and support for land-use activities. Please consider clarifying whether CTS is conditional on conservation, and, if not, frame it as "incentive-based conservation" rather than PES.
14. Please clarify the wording around the outcome variable: forest cover change, not deforestation per se. The text alternates between "deforestation/degradation" and "forest cover change." Please define clearly:
    (a) Is the outcome the forest cover proportion per cell?
    (b) Is "degradation" included as forest loss or treated separately?
    (c) If SIAT-AC distinguishes degradation, are you combining both or focusing only on deforestation?
15. Please clarify how you handle the overlap between programs.
    (a) Why were cells >40% (and not 20% or any overlap) chosen for baseline?
    (b) Is the overlap exclusion based on forest area or cell area (both are mentioned)?
16. In the limitations section, please briefly state whether missing illegal-road data is likely to bias estimates. For example, if treated sites are more remote/have less road access, or if roads are correlated with targeting.

Results

17. Figures 6–9 are informative but dense. Increasing font size and clearly labeling ATT units (percentage points vs. proportions) would improve readability. Also, consider adding sample sizes (N treated/N control) and units (percentage points vs. proportions) where applicable.
18. Please define outcome units consistently: state once whether ATT is in percentage points of forest cover, proportion of cell forested, or deforestation rate, and use that language consistently throughout text, tables, and captions.
19. Clarify the "three times lower" statement at the beginning of the results. Specify the baseline rate and the exact comparison period (for example: "0.28% vs X% in matched controls, averaged over YYYY–YYYY").
20. In the "Impact Analysis" below Fig. 5, you mention "three times more significant". Does this refer to "larger in magnitude" or "larger estimated effect"? If so, please consider rewording since "significant" can be misunderstood as statistical significance rather than size.

Discussion and Conclusion

21. Clearly separate empirical findings from policy implications, and specify the contextual conditions (tenure insecurity, cattle expansion, post-conflict governance) under which the results are generalizable, or where and when these findings are likely to apply.
22. It is noted that CTS shows a larger estimated annualized effect, but that this does not imply "better" performance. Consider combining relevant limitations into a short, explicit paragraph that explains why differences in estimated magnitudes should not be interpreted as "CTS being better than IFA", highlighting differences in:
    (a) Targeting and baseline deforestation risk
    (b) Incentive type and conditionality (cash vs. technical assistance/land-use planning)
    (c) Exposure length and timing of cohorts, and
    (d) Broader governance and program objectives.
23. The statement linking the Guaviare case to the United Nations Tropical Forest Forever Facility would benefit from stronger justification and more citations. Explicitly articulate which elements of the local evidence (differences in targeting, incentive structures, governance arrangements, and robustness to trend deviations) inform broader design principles for international forest finance. Please also consider situating this claim within the existing literature on jurisdictional and nested REDD+ and multi-level governance to bridge the empirical, subnational findings to the proposed international policy relevance in a more evidence-based way.
