# Supplementary material for: Incentives are effective but insufficient to halt smallholder deforestation in the Colombian Amazon
Source: PLoS One. 2026 Sep 23;21(9):e0340539. doi: 10.1371/journal.pone.0340539 (PMC13600516; doi:10.1371/journal.pone.0340539)
Supplement: Supplementary file 2 [file pone.0340539.s006.docx]

PONE-D-25-67913

Incentives are effective but insufficient to halt smallholder deforestation in the Colombian Amazon

Dear Editor and Reviewers:

Please find our response to each of your comments and suggestions in the Table below. We hope you find them satisfactory.

**Deadline 25th April**

**Reviews - what, who, how**

| **EDITOR** |  |
| --- | --- |
| What | How |
| Please ensure that your manuscript meets PLOS ONE's style requirements, including those for file naming. | Title page improved following provided guidelines. |
| Please note that PLOS One has specific guidelines on code sharing for submissions in which author-generated code underpins the findings in the manuscript. In these cases, we expect all author-generated code to be made available without restrictions upon publication of the work. Please review our guidelines at https://journals.plos.org/plosone/s/materials-and-software-sharing#loc-sharing-code and ensure that your code is shared in a way that follows best practice and facilitates reproducibility and reuse. | The author-generated R code can be found here: https://doi.org/10.6084/m9.figshare.32143840 |
| Thank you for stating the following financial disclosure:  “Spanish Ministry of Science and Innovation (PID2019-109758GB-I00 / AEI / 10.13039/501100011033) “María de Maeztu” Program for Units of Excellence of the Spanish Ministry of Science, Innovation and Universities (CEX2024-001506-M funded by MICIU/AEI/10.13039/501100011033)”  Please state what role the funders took in the study.  If the funders had no role, please state: "The funders had no role in study design, data collection and analysis, decision to publish, or preparation of the manuscript."  If this statement is not correct you must amend it as needed.  Please include this amended Role of Funder statement in your cover letter; we will change the online submission form on your behalf. | Text inserted in the re-submission cover letter. |
| In the online submission form, you indicated that your data is available only on request from a third party. Please note that your Data Availability Statement is currently missing [the name of the third party contact or institution / contact details for the third party, such as an email address or a link to where data requests can be made]. Please update your statement with the missing information. | We have included a data availability statement as requested. It reads as follows:  " The data underlying the findings of this study were derived from publicly available datasets (see Table 3), with the exception of land-use polygons associated with IFA and CTS participants, which cannot be publicly disclosed due to third-party restrictions. Researchers who meet the criteria for access to confidential data may request these data directly from the Government of Colombia’s Visión Amazonía REDD+ program (contact: visionamazonia@minabiente.gov.co) and from ONF-Andina (contact: abille@onfandina.com). The authors did not receive any special access privileges to government-held data. Access to ONF-Andina data was granted under a Memorandum of Understanding, which ensured the confidential handling of shared polygon data and required the authors to provide ONF-Andina with the research findings upon publication. The author-generated R code for this article can be found here: https://doi.org/10.6084/m9.figshare.32143840" |
| Please ensure that you include a title page within your main document. You should list all authors and all affiliations as per our author instructions and clearly indicate the corresponding author. | Addressed. |
| Please ensure that you refer to Figure 5 in your text as, if accepted, production will need this reference to link the reader to the figure. | Addressed. |
| We note that Figure 3 & SI Fig3 in your submission contain [map/satellite] images which may be copyrighted. All PLOS content is published under the Creative Commons Attribution License (CC BY 4.0), which means that the manuscript, images, and Supporting Information files will be freely available online, and any third party is permitted to access, download, copy, distribute, and use these materials in any way, even commercially, with proper attribution. For these reasons, we cannot publish previously copyrighted maps or satellite images created using proprietary data, such as Google software (Google Maps, Street View, and Earth). For more information, see our copyright guidelines: http://journals.plos.org/plosone/s/licenses-and-copyright.  We require you to either (1) present written permission from the copyright holder to publish these figures specifically under the CC BY 4.0 license, or (2) remove the figures from your submission:   a. You may seek permission from the original copyright holder of Figure 3 & SI Fig3 to publish the content specifically under the CC BY 4.0 license.  We recommend that you contact the original copyright holder with the Content Permission Form (http://journals.plos.org/plosone/s/file?id=7c09/content-permission-form.pdf) and the following text:  “I request permission for the open-access journal PLOS ONE to publish XXX under the Creative Commons Attribution License (CCAL) CC BY 4.0 (http://creativecommons.org/licenses/by/4.0/). Please be aware that this license allows unrestricted use and distribution, even commercially, by third parties. Please reply and provide explicit written permission to publish XXX under a CC BY license and complete the attached form.”  Please upload the completed Content Permission Form or other proof of granted permissions as an "Other" file with your submission.  In the figure caption of the copyrighted figure, please include the following text: “Reprinted from [ref] under a CC BY license, with permission from [name of publisher], original copyright [original copyright year].”   b. If you are unable to obtain permission from the original copyright holder to publish these figures under the CC BY 4.0 license or if the copyright holder’s requirements are incompatible with the CC BY 4.0 license, please either i) remove the figure or ii) supply a replacement figure that complies with the CC BY 4.0 license. Please check copyright information on all replacement figures and update the figure caption with source information. If applicable, please specify in the figure caption text when a figure is similar but not identical to the original image and is therefore for illustrative purposes only.  The following resources for replacing copyrighted map figures may be helpful:  USGS National Map Viewer (public domain): http://viewer.nationalmap.gov/viewer/  The Gateway to Astronaut Photography of Earth (public domain): http://eol.jsc.nasa.gov/sseop/clickmap/  Maps at the CIA (public domain): https://www.cia.gov/library/publications/the-world-factbook/index.html and https://www.cia.gov/library/publications/cia-maps-publications/index.html  NASA Earth Observatory (public domain): http://earthobservatory.nasa.gov/  Landsat: http://landsat.visibleearth.nasa.gov/  USGS EROS (Earth Resources Observatory and Science (EROS) Center) (public domain): http://eros.usgs.gov/#  Natural Earth (public domain): [http://www.naturalearthdata.com/](http://track.editorialmanager.com/CL0/http:%2F%2Fwww.naturalearthdata.com%2F/1/010f019cdb45d62c-040f6433-6e5f-4e0a-b140-bdc1938f3512-000000/bQRLu3Rr7Din_q9GdmR2XGbRoW5BR4yfyuT5otYtzLo=253) | To develop the figures of the Manuscript we used shapefiles from IGAC and SIAT-AC and these are available in these institutions' websites and they are publicly available. As such, they can be published under CCBY 4.0. Please see above our data availability statement. |
| Please include captions for your Supporting Information files at the end of your manuscript, and update any in-text citations to match accordingly. Please see our Supporting Information guidelines for more information: http://journals.plos.org/plosone/s/supporting-information. | Addressed. |
| **REVIEWER 1** |  |
| **INTRODUCTION** |  |
| The manuscript presents a methodologically rigorous evaluation of two initiatives, and the data and analyses support the conclusions. The statistical approaches are applied carefully and transparently. The manuscript is well written and clear. However, some improvements will strengthen the manuscript, including: (1) clarifying access to third-party datasets in the Data Availability Statement, (2) specifying whether staff interviews required IRB review, (3) ensuring consistent definition and labeling of outcome variables and ATT units across the results and figures, and (4) more explicitly grounding the broader policy implications in the Discussion. | We are grateful to the reviewer’s encouraging praise. We are also grateful for all the constructive comments provided below, which we have hopefully addressed as expected. |
| The data availability statement indicates that some data are held by third parties. It would be helpful to provide links or additional information for these sources. | As per above |
| The ethics statement is listed as N/A. However, the authors report "*interviews with staff from IFA and CTS to gain access to key documentation and obtain information about program goals, eligibility rules, and implementation practices*", which needs clarification regarding an IRB (Institutional Review Board) and/or exemption. | Thank you so much for this comment. The IRB at UAB deems exempt from ethical review interviews with professionals talking freely about their experience, and always that the UAB's Code of Good Practice in Research is followed (free consent, confidentiality, opt-out, etc.). The IRB is currently publishing this information on the web, along with other reasons for ethical exemption, but this process will take some time. Meanwhile, you can contact the CERec office at cerec@uab.cat or, if necessary, we can apply for an exemption letter.  Notwithstanding, we have improved the text in the main manuscript to make it much clearer that we did not conduct formal interviews as such, but rather obtained basic information and data from key staff in Visión Amazonía and ONF. The text now reads as follows:  “To obtain basic information about the IFA and CTS goals and to access key data, we contacted key staff from *Visión Amazonía* and ONF-Andina. They provided us with shapefiles for each participant’s landholding, which also included the total area of protected forest under each contract. For CTS, we additionally obtained shapefiles for some contracts that were signed but never implemented, or that were discontinued shortly after signing because landowners chose not to continue enrolled. We used these cases as a placebo test in our estimations.” |
| Explicitly state research questions/objectives of the study. A summary table linking research questions, estimates, data sources, and figures could improve accessibility. | Thank you for this insightful comment. We have improved the key paragraph in the Introduction as follows, but we have not developed the suggested Table to avoid increasing the number of Tables and Figures, which is already high. You will also note that the text expands on the characterisation of the two studied programs, in order to address one of your comments further below.  “In this article, we analyse the success of two PES initiatives in achieving their goal of deterring deforestation in the department of Guaviare, Colombia. The *Incentivo Forestal Amazónico* (IFA) program provides monetary payments for forest conservation and is implemented by the Colombian government under the broader policy framework of *Visión Amazonía*, an ambitious REDD+ Early Movers program funded by the governments of Norway, the United Kingdom, and Germany (<https://visionamazonia.minambiente.gov.co/>). Launched in 2018, the program is jointly managed by the NGO Más Bosques, three environmental authorities of the Colombian Amazon region, and the Ministry of Environment and Sustainable Development, each assuming distinct roles. IFA is a key pillar of the REDD+ strategy and operates as a conventional PES intervention across five departments of the country's Amazon region (Guaviare, Caquetá, Putumayo, Meta, Amazonas), where direct cash payments are provided to households in exchange for conservation commitments over clearly defined forest areas. The *Caminemos Territorios Sostenibles* (CTS) project, in turn, was implemented between 2018 and 2022 by the NGO ONF-Andina along the agricultural frontier of Guaviare. It combined both cash and in-kind land-use planning technical assistance, with the former being tied to the amount and type of conservation and restoration activities to be performed on the farm (see *Materials and methods* below).    For both IFA and CTS, we specifically ask: how do government-led REDD+ payments and NGO-provided incentives contribute to reducing deforestation? How do distinct design and implementation features affect avoided deforestation outcomes? Understanding the effectiveness of current PES policies and projects is particularly urgent in a region where new actors are grabbing forest land for cattle ranching, coca cultivation, and other extractive activities such as gold mining (Armenteras et al., 2019; Murillo-Sandoval et al., 2023; Prem et al., 2020; Van Dexter & Visseren-Hamakers, 2020). It is also important to examine the differing impacts of two contrasting PES designs: one focused on payments only and broad in reach with another providing land-use planning assistance as well as economic support in a more circumscribed area of the agricultural frontier. Comparing these approaches can provide important insights into what works best and why for reducing deforestation in the study region.  To our knowledge, only one study has assessed the ecological effectiveness of PES in the Colombian Amazon (Econometría, 2025). Three additional studies have examined environmental outcomes of PES in Colombia, although all are limited to silvopastoral PES schemes in the Quindío and Valle del Cauca regions (Calle, 2020; Pagiola et al., 2016; Pagiola & Ríos, 2013). None addresses the two gaps we seek to fill in this article. First, we examine how well two PES programs which overlap in the same deparment perform and, second, how such performance is influenced by the programs' design features. Elsewhere, research has explored how different conservation and social policy instruments interact within the same landscape, for example protected areas and PES (Montoya-Zumaeta et al., 2019; Robalino et al., 2015; Sims & Alix-García, 2017) or PES and anti-poverty programs (Izquierdo-Tort, 2020). In tropical frontier regions like Guaviare, where deforestation pressure is acute, it is common for multiple conservation projects led by public agencies, civil society, and private actors to coexist (Furumo & Lambin, 2020). While this proliferation of environmental programs can help fill governance gaps and mobilize rapid action, it can also create inefficiencies and coordination challenges (Lambin et al., 2014). Analysing the overlapping but contrasting designs of IFA and CTS therefore enables us to assess the combined effects of policy mixes and to compare how differences in incentive structure, conditionality, and governance arrangements influence conservation outcomes. Addressing these interactions is especially important in light of emerging international frameworks, such as the Tropical Forest Forever Facility, which aim to scale up incentive-based forest conservation in complex, multi-actor settings.” |
| Please clarify the connection between:  The conceptual logic from PES theory => expected mechanisms => testable expectations  This clarification will help explain why comparing cash transfers (IFA) with technical assistance/land-use planning (CTS) is theoretically meaningful rather than only empirically convenient. | Please see the response to the following comment. We have added a new paragraph that clarifies the PES conceptual logic and expected outcomes. |
| The introduction summarizes PES generally and then jumps to "mixed evidence." Consider adding 2–3 sentences that explain mechanisms by which incentives should reduce deforestation (increasing relative returns to conservation, adoption of lower-deforestation practices, changing land-use planning/monitoring), and why those may fail (high opportunity costs, weak enforcement/tenure, powerful external actors). | Thank you for this comment. We have improved the correspondent paragraph in the Introduction by adding the following text:  “A growing body of research has focused on assessing PES effectiveness and the factors that explain their success or failure. PES can reduce deforestation through several mechanisms that can act synergistically: by increasing the relative returns to forest conservation compared to alternative land uses, by encouraging the adoption of low-deforestation productive practices, and by strengthening land-use planning, collective environmental governance, monitoring, and compliance. PES are expected to change land-use behaviour by increasing the financial returns of conservation, supporting the intensification of more sustainable agriculture, or strengthening local conservation institutions. However, as noted earlier, these mechanisms do not always operate in conjunction or perform as intended. Their effects may be undermined by high opportunity costs of forest conservation, weak enforcement and insecure land tenure, or strong pressures from external actors such as cattle ranchers, agro-industrial interests, or illegal economies.” |
| It is not clear why the two initiatives under analysis are labeled "PES". CTS is described as technical assistance and land-use plans (in-kind payments). This classification may fit "incentive-based conservation" broadly, but it might not be considered PES in the strict conditional-payment sense. Please justify the classification (conditional incentives linked to conservation commitments/land-use plans) or adjust the language to "incentive-based conservation programs". | We are thankful for this comment. It made us realise that we had probably not explained the design features of both studied programs well enough. Therefore, as noted in one of the earlier comments, we improved the description of both programs in the Introduction section.  Seemingly, we have improved the abstract to make clear that CTS also includes financial compensation. |
| Consider elaborating on the research gap. As it is, the introduction only states that three studies analyze PES effectiveness in Colombia's Amazon and none examines overlapping initiatives. It is a useful and informative gap, but it would be stronger if you specified what is unknown:  (a) Is the gap mainly due to differences in comparative effectiveness between different incentive types (cash vs in-kind/TA)?  (b) Or policy-mix interactions/overlap (multiple programs in the same landscape)? | Thank you for this comment. We have now made clearer in the Introduction the two research gaps addressed in the paper. We now state:  “To our knowledge, only one study has assessed the ecological effectiveness of PES in the Colombian Amazon (Econometría, 2025). Three additional studies have examined environmental outcomes of PES in Colombia, although all are limited to silvopastoral PES schemes in the Quindío and Valle del Cauca regions (Calle, 2020; Pagiola et al., 2016; Pagiola & Ríos, 2013). None addresses the two gaps we seek to fill in this article, i.e. how well two PES programs which overlap in the same deparment perform and, how such performance is influenced by the programs' design and implementation features.” |
| I suggest avoiding broad quantitative statements without context/citations. For example, "…can globally release as much CO₂ _annually as all EU countries  combined". This sentence may be directionally right, but it needs to be contextualized (year? includes degradation? includes soil or other sectors?). Consider including a more standard statement on global emissions from deforestation/land-use change (with an updated citation if needed). | Thank you. We have improved the opening statement, to be more precise and to provide a more updated reference. This now reads as follows:  “Deforestation is one of the most pressing environmental challenges of our time. It contributes to biodiversity loss and represents the main driver of land-use related emissions, at around 3.9 GtCO2 per year over the 2015-2024 period (Friedlingstein et al., 2025).” |
| The introduction would benefit from a short paragraph situating IFA within the historical evolution of Colombia's national REDD+ framework, including the timeframe, to help explain the policy logic behind the program's design and targeting. | To address this comment while avoiding an increase in the length of the manuscript, we have moved some of the information we formerly had in the program’s description section to the Introduction, in order to provide more contextual information there. The text referred to IFA in the Introduction now reads as follows:  “In this article, we analyse the success of two PES initiatives in achieving their goal of deterring deforestation in the department of Guaviare, Colombia. The *Incentivo Forestal Amazónico* (IFA) program provides monetary payments for forest conservation and is implemented by the Colombian government under the broader policy framework of *Visión Amazonía*, an ambitious REDD+ Early Movers program funded by the governments of Norway, the United Kingdom, and Germany (<https://visionamazonia.minambiente.gov.co/>). Launched in 2018, the program is jointly managed by the NGO Más Bosques, three environmental authorities of the Colombian Amazon region, and the Ministry of Environment and Sustainable Development, each assuming distinct roles. IFA is a key pillar of the REDD+ strategy and operates as a conventional PES intervention across five departments of the country's Amazon region (Guaviare, Caquetá, Putumayo, Meta, Amazonas), where direct cash payments are provided to households in exchange for conservation commitments over clearly defined forest areas. The *Caminemos Territorios Sostenibles* (CTS) project, in turn, was implemented between 2018 and 2022 by the NGO ONF-Andina along the agricultural frontier of Guaviare. It combined both cash and in-kind land-use planning technical assistance, with the former being tied to the amount and type of conservation and restoration activities to be performed on the farm (see *Materials and methods* below).” |
| **MATERIALS AND METHODS** |  |
| Fix typos: enrolment, enrol. | Typos have been amended throughout the manuscript. We sticked to "enroll" as for American English, which is accepted in PLOS One. |
| I suggest defining acronyms once and consistently: CPTA/CPT, DR, CSA, etc. are used heavily. Please add a short "Notation/abbreviations" sentence or ensure consistent naming ("conditional parallel trends assumption" vs "CPTA"). | Checked, all the abbreviations have already been corrected. |
| The context section is strong, and the historical narrative is informative. However, some details (rubber company history, colonization waves) may be more appropriate for the Introduction/Background or Discussion, unless they are directly linked to the identification or selection of covariates. | We are grateful for this comment. Since the paper is already loaded with significant contextual information that is important to understand our choice of variables or to frame our discussion, we decided to delete the full paragraph that explained Guaviare’s colonization period because such explanation was not analytically relevant. |
| Please clarify the "treatment" concept. IFA appears closer to classic conditional payments, and CTS is a bundle of planning and support for land-use activities. Please consider clarifying whether CTS is conditional on conservation, and, if not, frame it as "incentive-based conservation" rather than PES. | Thank you for this comment. We have clarified what it is meant by PES in this article, which matches broad definition of the term that consider it an approach that can combine, in specific contexts, both payments and in-kind or non-monetary incentives. We have also clarified the nature of the incentive for both IFA and CTS, in several passages of the re-submitted manuscript. We copy below those which we believe contribute to address this comment.  Introduction [PES description]  “Since the early 2000s, Payments for Ecosystem Services (PES) programs have been adopted globally as a key instrument to promote forest management, restoration, and conservation. PES consist of monetary -and sometimes also in-kind- incentives provided to landowners and communities in the form of short-term contracts (i.e. 3-5 years), conditional on undertaking certain activities that should protect standing forests or enhance the provision of ecosystem services. They have gained popularity because they can provide both conservation and socio-economic benefits (Bulte et al., 2008) and, as of today, there are hundreds of PES programs and projects distributing dozens of billions of dollars in annual transactions (Salzman et al., 2018). These PES programs vary widely in their design and implementation aspects, including their goals –ecosystem conservation or provision of specific ecosystem services (e.g. carbon, water, biodiversity)–, geographic scale –local, subnational, national, international–, participating actors –governments, NGOs, private companies, multilateral organizations–, and contract design features –e.g. incentive structure, targeting, enforcement– (Wunder et al. 2018).  A key innovation of PES has been their conditionality and voluntary character, which reduced concerns about lack of retribution and exclusion that were common in prior command-and-control instruments such as protected areas (Wunder, 2013). Directly linking the provided incentives to specific outcomes or land-use activities is also expected to address common shortcomings of earlier, more indirect approaches like Integrated Conservation and Development Projects (ICDPs) (Ferraro and Kiss, 2002). Cash payments or in-kind benefits like seedlings, equipment, and training can help increase the competitiveness of forest conservation relative to alternative land uses that are more economically profitable in the absence of PES (Engel et al. 2008; Wunder, 2013).”  Introduction [IFA and CTS description]  “In this article, we analyse the success of two PES initiatives in achieving their goal of deterring deforestation in the department of Guaviare, Colombia. The *Incentivo Forestal Amazónico* (IFA) program provides monetary payments for forest conservation and is implemented by the Colombian government under the broader policy framework of *Visión Amazonía*, an ambitious REDD+ Early Movers program funded by the governments of Norway, the United Kingdom, and Germany (<https://visionamazonia.minambiente.gov.co/>). Launched in 2018, the program is jointly managed by the NGO Más Bosques, three environmental authorities of the Colombian Amazon region, and the Ministry of Environment and Sustainable Development, each assuming distinct roles. IFA is a key pillar of the REDD+ strategy and operates as a conventional PES intervention across five departments of the country's Amazon region (Guaviare, Caquetá, Putumayo, Meta, Amazonas), where direct cash payments are provided to households in exchange for conservation commitments over clearly defined forest areas. The *Caminemos Territorios Sostenibles* (CTS) project, in turn, was implemented between 2018 and 2022 by the NGO ONF-Andina along the agricultural frontier of Guaviare. It combined both cash and in-kind land-use planning technical assistance, with the former being tied to the amount and type of conservation and restoration activities to be performed on the farm (see *Materials and methods* below).”  Materials and methods [IFA and CTS description – See also Table 1]  “*The Incentivo Forestal Amazónico (IFA)*  As noted in the Introduction, the IFA program aims to reduce deforestation by providing monetary incentives to smallholders for forest conservation. Between 2020 and 2022, it had enrolled 109,425 hectares of primary forest across its five targeted departments. In Guaviare, implementation in Guaviare began in 2020 and was scaled up from 2021 onwards. The pilot phase in 2020 included 194 participants; however, contracts lacked clearly defined conservation areas, payment rules were still under development, and compliance monitoring was weak. For these reasons, we excluded the pilot phase from our analysis. By early 2023, 516 households in 61 communities across the department’s four municipalities had joined IFA, enrolling landholdings for a total of 25,862 hectares and placing 16,615 hectares under forest conservation contracts (Table 1).  To be eligible for enrollment, the smallholder's property had to have at least 30% of its total area under forest cover and contain a minimum of 10 hectares of forest. This implied a minimum total property size of 33.3 hectares. This rule was established to encourage the participation of smaller plots, particularly in Putumayo, where average plot sizes are smaller than in Guaviare or Meta. The program aimed to enrol all forested areas within a family property landholding; however, for smaller properties, participants could freely decide how much forest to include in the contract, provided they met the 10-hectare minimum. The enrolled area determined the payment received: 2.4 million COP per year for enrolling 30-59% of the forested area, 3 million COP for 60–74%, and 3.6 million COP for more than 75%. Payments were delivered in four instalments via bank transfer, and contracts lasted approximately three years. Compliance was measured using satellite monitoring, considering the early deforestation alerts issued by the IDEAM, and complemented with field visits, when possible.  *Caminemos Territorios Sostenibles (CTS)*  The five-year CTS project implemented by ONF-Andina was aimed at developing sustainable land-use plans for family farms along the agricultural frontier of Guaviare, i.e. in the northwestern part of the department, in order to encourage on-farm conservation and restoration activities, as well as the conversion of extensive cattle grazing into a silvopastoral system. To be eligible for enrollment, each household had to characterize and map its landholding and develop the correspondent sustainable land-use plan. The technical and financial support provided depended on the ecological characteristics of the farm, the number of conservation and restoration activities to be implemented, and the investment and goods required (e.g. barbwire, seedlings, waterholes, etc.) to move towards a silvopastoral system. The NGO assigned each enrolled household a score ranging from 0 to 100, and the payment level depended on the household’s capacity to cover the costs of the planned activities, the degree of ambition and viability of such activities, the amount of forests on the farm, and the household’s past deforestation behaviour. As a result, payments per household oscillated between 7 and 12 million COP over the project's timeframe. Compliance was monitored through CTS’s own platform, Forland, which used satellite imagery to generate monthly deforestation alerts (ONF International, 2025). A total of 400 participants across 40 communities enrolled gradually between June 2018 and June 2019, covering 27,606 hectares and placing 12,301 hectares of these under conservation. Each household was enrolled for approximately three to four years (Table 1).” |
| Please clarify the wording around the outcome variable: forest cover change, not deforestation per se. The text alternates between "deforestation/degradation" and "forest cover change." Please define clearly:  (a) Is the outcome the forest cover proportion per cell?  (b) Is "degradation" included as forest loss or treated separately?  (c) If SIAT-AC distinguishes degradation, are you combining both or focusing only on deforestation? | Thank you for this comment. Let us clarify:   (a) The outcome is indeed forest proportion per cell. (b) When using the JRC, the undisturbed forest cover loss takes into account forest degradation, and for all forest cover loss, only deforestation is taken into account. When using SIAT-AC data, we do not take into account forest degradation since this database does not distinguish deforestation from degradation. (c) The map we use for SIAT-AC does not distinguish degradation from deforestation but only provides binary information (Forest / Not forest) |
| Please clarify how you handle the overlap between programs.  (a) Why were cells >40% (and not 20% or any overlap) chosen for baseline?  (b) Is the overlap exclusion based on forest area or cell area (both are mentioned)? | Thank you. Let us clarify:   (a) We chose this threshold to reduce the risk of bias while keeping an important number of observations in the analysis. Because this threshold is arbitrary, however, we used other thresholds as robustness checks and we observed that these other tested thresholds did not affect the impact estimate nor its significance. (b) The overlap is based on forest area: it is now amended in the resubmitted manuscript. |
| In the limitations section, please briefly state whether missing illegal-road data is likely to bias estimates. For example, if treated sites are more remote/have less road access, or if roads are correlated with targeting. | Thank you for this comment, the illegal roads data might indeed bias our estimates and we now explain this better in the Internal and external validity sub-section. This now reads as follows:  “The assumption least likely to hold in our analysis is the CPTA, as our list of matching covariates was not exhaustive. We were unable to map and control for illegal roads, which may have led to an underestimation of the treatment effect. Deforestation risk is often higher in properties closer to illegal roads and the probability to enrol in the program may also be higher along such roads because of increased accessibility and access to policy programs and projects. We were also unable to match on households’ socio-economic characteristics, as such data were unavailable for non-enrolled households and parcels in our study region.  Because of such data availability restrictions, we ran the analysis only for a sub-region of Guaviare where deforestation risk is expected to be higher. Therefore, our results for the IFA program may not be valid for the entirety of Guaviare. In this case, the overall effect might be lower than the effect measured for the impact analysis region, since some of the areas falling outside the impact analysis area are rather remote and deforestation risk may be lower.” |
| **RESULTS** |  |
| Figures 6–9 are informative but dense. Increasing font size and clearly labeling ATT units (percentage points vs. proportions) would improve readability. Also, consider adding sample sizes (N treated/N control) and units (percentage points vs. proportions) where applicable. | We have improved all figures as requested. |
| Please define outcome units consistently: state once whether ATT is in percentage points of forest cover, proportion of cell forested, or deforestation rate, and use that language consistently throughout text, tables, and captions. | Thank you for this remark, it was indeed unclear in the last version that we use share of enrolled areas expressed in % as unit for the ATT. It has been corrected in the manuscript and in the figures. |
| Clarify the "three times lower" statement at the beginning of the results. Specify the baseline rate and the exact comparison period (for example: "0.28% vs X% in matched controls, averaged over YYYY–YYYY"). | Thank you for the comment, a clarification has been made in that sense. The new text now reads as follows:  “On average, forest cover is 2.9% higher in areas enrolled in the IFA program compared to non-enrolled, control areas facing similar deforestation pressures (95% confidence interval [1.51:4.38]) (Fig 8a). We therefore estimate that approximately 276 ha of forest cover were preserved by the program between 2021 and 2022. The magnitude of the effect is four times greater in the CTS project area, where forest cover is 12% higher in enrolled areas compared to non-enrolled control areas (CI: [8.33: 15.6]) (Fig 8b). This represents approximately 1,130 ha of avoided deforestation in the CTS targeted area between 2019 and 2022.” |
| In the "Impact Analysis" below Fig. 5, you mention "three times more significant". Does this refer to "larger in magnitude" or "larger estimated effect"? If so, please consider rewording since "significant" can be misunderstood as statistical significance rather than size. | Thank you for underlining the lack of clarity. The sentence has been changed as shown above. |
| **DISCUSSION** |  |
| Clearly separate empirical findings from policy implications, and specify the contextual conditions (tenure insecurity, cattle expansion, post-conflict governance) under which the results are generalizable, or where and when these findings are likely to apply. | We have improved the discussion a bit, moved some text around, and created two separate sub-sections that now neatly distinguish between empirical findings and policy implications. Please look at the “manuscript_with track changes” file to see all the changes we made in this section. We feel that the discussion is now much stronger and clear. |
| It is noted that CTS shows a larger estimated annualized effect, but that this does not imply "better" performance. Consider combining relevant limitations into a short, explicit paragraph that explains why differences in estimated magnitudes should not be interpreted as "CTS being better than IFA", highlighting differences in:  (a)  Targeting and baseline deforestation risk  (b)  Incentive type and conditionality (cash vs. technical assistance/land-use planning)  (c)  Exposure length and timing of cohorts, and  (d)  Broader governance and program objectives. | Thank you for this suggestion. The sub-section on empirical findings now ends with the following short paragraph:  “Taken together, these findings suggest that IFA and CTS have both had a positive but modest conservation impact, yet the limitations in the number of matching covariates employed and the spatial reach of our impact analysis area may have led to an overestimation of their annualized effect on avoided deforestation. It is also important to stress that their distinct design and implementation features, including different targeting and baseline deforestation risk, type of incentive (only cash vs. cash, technical assistance and land-use planning), exposure length and timing of cohorts, as well as broader objectives, would make such comparison futile. Instead, we argue that the two studied programs have reinforced one another in a region characterized by a weak, often conflict-ridden, land governance context (Lambin et al., 2014). Future research should investigate whether the IFA annual effect has increased or decreased over time as more participants joined or abandoned the program and, concurrently, compare these effects with the levels of permanence observed in previously treated CTS areas.” |
| The statement linking the Guaviare case to the United Nations Tropical Forest Forever Facility would benefit from stronger justification and more citations. Explicitly articulate which elements of the local evidence (differences in targeting, incentive structures, governance arrangements, and robustness to trend deviations) inform broader design principles for international forest finance. Please also consider situating this claim within the existing literature on jurisdictional and nested REDD+ and multi-level governance to bridge the empirical, subnational findings to the proposed international policy relevance in a more evidence-based way. | As noted above, we think we have significantly improved the Policy implications sub-section of the Discussion. |
| **REVIEWER 2** |  |
| The manuscript contributes to the increasing literature on impact evaluations of forests conservation policies by assessing and comparting two incentive-based initiatives implemented simultaneously at some extent in an specific context of the Colombian Amazon. The comparative perspective in this regard represents a valuable advance in this literature and could provide important insights to increase our understanding about what does (and does not) works in forest conservation. However, there are some important improvements that are required to be addressed before being accepted for publication. | Thank you for this encouraging and positive comment. |
| My main concern is regarding the novelty of the research and its results. Although empirical results from the analysis are valuable and worthy; in the end, it reveals something that already has been widely discussed in the forest conservation literature: that incentive-based interventions, in most of the cases, yield positive yet modest deforestation outcomes. In this sense, I suggest you to discuss more explicitly across the manuscript (especially in the introductory and discussion Sections) what are the meaningful contributions that your analysis makes not only in terms of the empirical results that offer but also in relation to the theoretical and practical insights that could guide future research or practice in designing effective forest conservation policies. | Thank you so much for this comment. This was somewhat a concern for another reviewer, and we have tried to attend for this suggestion in different ways.  First, we have made our contribution clearer in the Introduction, by providing the research questions that are addressed in the paper and why they matter, and by making much more explicit the research gaps we address.  Second, we have re-organised the discussion to distinguish more neatly between empirical findings and policy implications, and in so doing adding specific paragraphs and statements about why the programs’ analysed cannot be compared per se but draw instead important lessons on the importance of implementing conservation instruments that are synergistic and overlap in regions with high deforestation trends and weak governance. |
| The Methods Section is explained in great detail, however there are some methodological choices that seemingly are questionable. First, it is not clear enough whether grids used as controls cover only cover eligible areas (i.e farmers' managed lands) or did you the whole "impact analysis area" that, I suppose, include areas with differentiated tenure regimes (i.e. collective lands), or even, not defined property rights. If that is the case, this could imply that some unobserved differences were not completely controlled for. How did you deal with this potential issue? | Thank you for this comment. Guaviare is a region where land tenure rights are not clearly defined so no regional land registry exists. Therefore, the cell grids used as control encompass all forest cover in the area for which we have data from SIAT-AC, and we do not (could not) control for land tenure type.  If there is potential for unobserved differences not being controlled for, this risk seems quite low for two reasons.   - First, there are no important differences in terms of land tenure in the region, it is mostly individual (except indigenous reserves that we take into account as covariates) the only difference is between occupants and legal landholders. - Second PES programs accepted both legal landholders and occupants without making differences, thus decreasing the risk of enrollment being correlated with the nature of property rights, and therefore the risk of bias in our estimate. - Finally, it is most likely that our covariates already capture most of the unobserved differences: legally owned land are usually closer to "veredas" centers and roads. |
| Also, in relation to the methodological Section, while it is remarkable you apply a newer approach for testing the parallel trends assumption (PTA), in the interest of being transparent in regard to your results and their robustness, I would suggest that you could also perform and show the results of traditional either graphical or placebo PTA tests (at least as Supplementary Material). | Thank you for this comment, indeed the newer approach might make our methodology less transparent in regards to how we test the CPTA, and classical ways to test the CPTA such as graphical proof or placebo provide an insight easier to understand. Those two approaches are already visible in the graphs we use in the manuscript, namely:     - The placebo tests are already performed as part of the Callaway and Sant’anna "did package" since it computes the “treatment effect” before the treatment starts (this is what we see in Figure 8, where we can see that before the treatment, the estimates are not different from 0 for both programs at all time-periods). - Graphical CPTA: Since we rely on CPTA and not PTA (The parallel trend assumption is conditional on covariates being the same), we cannot provide a graphical proof as for more classical DiD settings. However, Figures 8 and 9 can be used as graphical tests of the CPTA. Figure 8 shows for each cohort of the IFA the difference in trends between treated and control before the treatment. It shows graphical evidence that there is no trend in the difference between treated and control before the treatment in each cohort. As for CTS, since the program only had one cohort, the Figure 8 event study is enough to provide graphical evidence of the CPTA. |
| Also, in this regard, would it be important to clarify whether and how did you control for spillovers in the evaluated outcome. Eventually, if you have not performed such an analysis, please elaborate the reasons of this decision. The analysis of potential spillovers could provide important insights in relation to the obtained results and potential mechanisms driving these. | Thanks for raising this crucial issue. The main risk of spillovers is on-site spillover since the programs register only the share of the property that is included in the conservation contract, or the land-use plan. However, we think that our identification strategy prevents this bias because we consider as treated the whole property and not just only the area registered as conserved. |
| It was not possible to combine existing socioeconomic data of the study area (maybe from primary or secondary sources) with the performed spatial analysis? Using this type of data to the analysis could bring important benefits to identify mechanisms driving difference in the interventions outcomes. In case this was not possible, at least needs to be listed as a potential limitation of the analyses and/or a potential future research. | Thank you for this remark. Unfortunately, the socio-economic data only exists at "vereda" level, which is not precise enough for our analysis. We added this remark in the *Internal and external validity* sub-section. |
| In addition to criteria for targeting areas, was there any other difference in the design of analyzed intervention that might have enable difference in outcomes. Besides those discussed already, the shorter duration of the IFA contracts relative to longer and apparently more the intensive CTS treatment did not have implications in their overall lower deforestation impacts? Might these differences also have implications in terms of the permanence of their outcomes? | Thank you for this comment. The discussion of the article has been re-organised as noted above, and the *Summary of findings* sub-section now discusses in more depth the design and implementation features that may influence the difference in outcome of the two programs. In this renewed sub-section, we have also noted that "Future research should investigate whether the IFA annual effect has increased or decreased over time as more participants joined or abandoned the program and, concurrently, compare these effects with the levels of permanence observed in previously treated CTS areas." |
