# Supplementary material for: Incentives are effective but insufficient to halt smallholder deforestation in the Colombian Amazon
Source: PLoS One. 2026 Sep 23;21(9):e0340539. doi: 10.1371/journal.pone.0340539 (PMC13600516; doi:10.1371/journal.pone.0340539)
Supplement: Peer review history

# Peer review: Decision Letter 0

**DOI:** 10.1371/journal.pone.0340539.r001

**Authors:** Rajan Parajuli

11 Mar 2026

Dear Dr. Corbera,

Thank you for submitting your manuscript to PLOS ONE. After careful consideration, we feel that it has merit but does not fully meet PLOS ONE’s publication criteria as it currently stands. Therefore, we invite you to submit a revised version of the manuscript that addresses the points raised during the review process.

Please submit your revised manuscript by Apr 25 2026 11:59PM. If you will need more time than this to complete your revisions, please reply to this message or contact the journal office at plosone@plos.org. When you're ready to submit your revision, log on to https://www.editorialmanager.com/pone/ and select the 'Submissions Needing Revision' folder to locate your manuscript file.

- A letter that responds to each point raised by the academic editor and reviewer(s). You should upload this letter as a separate file labeled 'Response to Reviewers'.
- A marked-up copy of your manuscript that highlights changes made to the original version. You should upload this as a separate file labeled 'Revised Manuscript with Track Changes'.
- An unmarked version of your revised paper without tracked changes. You should upload this as a separate file labeled 'Manuscript'.

If you would like to make changes to your financial disclosure, please include your updated statement in your cover letter. Guidelines for resubmitting your figure files are available below the reviewer comments at the end of this letter.

If applicable, we recommend that you deposit your laboratory protocols in protocols.io to enhance the reproducibility of your results. Protocols.io assigns your protocol its own identifier (DOI) so that it can be cited independently in the future. For instructions see: https://journals.plos.org/plosone/s/submission-guidelines#loc-laboratory-protocols. Additionally, PLOS ONE offers an option for publishing peer-reviewed Lab Protocol articles, which describe protocols hosted on protocols.io. Read more information on sharing protocols at https://plos.org/protocols?utm_medium=editorial-email&utm_source=authorletters&utm_campaign=protocols.

We look forward to receiving your revised manuscript.

Kind regards,

Rajan Parajuli

Academic Editor

PLOS One

Journal Requirements:

When submitting your revision, we need you to address these additional requirements.

1.Please ensure that your manuscript meets PLOS ONE's style requirements, including those for file naming. The PLOS ONE style templates can be found at

https://journals.plos.org/plosone/s/file?id=wjVg/PLOSOne_formatting_sample_main_body.pdf and

https://journals.plos.org/plosone/s/file?id=ba62/PLOSOne_formatting_sample_title_authors_affiliations.pdf.

2. Please note that PLOS One has specific guidelines on code sharing for submissions in which author-generated code underpins the findings in the manuscript. In these cases, we expect all author-generated code to be made available without restrictions upon publication of the work. Please review our guidelines at https://journals.plos.org/plosone/s/materials-and-software-sharing#loc-sharing-code and ensure that your code is shared in a way that follows best practice and facilitates reproducibility and reuse.

3. Thank you for stating the following financial disclosure:

“Spanish Ministry of Science and Innovation (PID2019-109758GB-I00 / AEI / 10.13039/501100011033)

“María de Maeztu” Program for Units of Excellence of the Spanish Ministry of Science, Innovation and Universities (CEX2024-001506-M funded by MICIU/AEI/10.13039/501100011033)”

Please state what role the funders took in the study.  If the funders had no role, please state: "The funders had no role in study design, data collection and analysis, decision to publish, or preparation of the manuscript."

If this statement is not correct you must amend it as needed.

Please include this amended Role of Funder statement in your cover letter; we will change the online submission form on your behalf.

4. In the online submission form, you indicated that your data is available only on request from a third party. Please note that your Data Availability Statement is currently missing [the name of the third party contact or institution / contact details for the third party, such as an email address or a link to where data requests can be made]. Please update your statement with the missing information.

5. Please ensure that you include a title page within your main document. You should list all authors and all affiliations as per our author instructions and clearly indicate the corresponding author.

6. Please ensure that you refer to Figure 5 in your text as, if accepted, production will need this reference to link the reader to the figure.

7. We note that Figure 3 & SI Fig3 in your submission contain [map/satellite] images which may be copyrighted. All PLOS content is published under the Creative Commons Attribution License (CC BY 4.0), which means that the manuscript, images, and Supporting Information files will be freely available online, and any third party is permitted to access, download, copy, distribute, and use these materials in any way, even commercially, with proper attribution. For these reasons, we cannot publish previously copyrighted maps or satellite images created using proprietary data, such as Google software (Google Maps, Street View, and Earth). For more information, see our copyright guidelines: http://journals.plos.org/plosone/s/licenses-and-copyright.

We require you to either (1) present written permission from the copyright holder to publish these figures specifically under the CC BY 4.0 license, or (2) remove the figures from your submission:

a. You may seek permission from the original copyright holder of Figure 3 & SI Fig3 to publish the content specifically under the CC BY 4.0 license.

We recommend that you contact the original copyright holder with the Content Permission Form (http://journals.plos.org/plosone/s/file?id=7c09/content-permission-form.pdf) and the following text:

“I request permission for the open-access journal PLOS ONE to publish XXX under the Creative Commons Attribution License (CCAL) CC BY 4.0 (http://creativecommons.org/licenses/by/4.0/). Please be aware that this license allows unrestricted use and distribution, even commercially, by third parties. Please reply and provide explicit written permission to publish XXX under a CC BY license and complete the attached form.”

Please upload the completed Content Permission Form or other proof of granted permissions as an "Other" file with your submission.

In the figure caption of the copyrighted figure, please include the following text: “Reprinted from [ref] under a CC BY license, with permission from [name of publisher], original copyright [original copyright year].”

b. If you are unable to obtain permission from the original copyright holder to publish these figures under the CC BY 4.0 license or if the copyright holder’s requirements are incompatible with the CC BY 4.0 license, please either i) remove the figure or ii) supply a replacement figure that complies with the CC BY 4.0 license. Please check copyright information on all replacement figures and update the figure caption with source information. If applicable, please specify in the figure caption text when a figure is similar but not identical to the original image and is therefore for illustrative purposes only.

The following resources for replacing copyrighted map figures may be helpful:

USGS National Map Viewer (public domain): http://viewer.nationalmap.gov/viewer/

The Gateway to Astronaut Photography of Earth (public domain): http://eol.jsc.nasa.gov/sseop/clickmap/

Maps at the CIA (public domain): https://www.cia.gov/library/publications/the-world-factbook/index.html and https://www.cia.gov/library/publications/cia-maps-publications/index.html

NASA Earth Observatory (public domain): http://earthobservatory.nasa.gov/

Landsat: http://landsat.visibleearth.nasa.gov/

USGS EROS (Earth Resources Observatory and Science (EROS) Center) (public domain): http://eros.usgs.gov/#

Natural Earth (public domain): http://www.naturalearthdata.com/

8. Please include captions for your Supporting Information files at the end of your manuscript, and update any in-text citations to match accordingly. Please see our Supporting Information guidelines for more information: http://journals.plos.org/plosone/s/supporting-information.

9. If the reviewer comments include a recommendation to cite specific previously published works, please review and evaluate these publications to determine whether they are relevant and should be cited. There is no requirement to cite these works unless the editor has indicated otherwise.

Additional Editor Comments (if provided):

Two reviewers provided detailed comments and suggestions which I wholly agree with. I encourage authors to revise your manuscript carefully and resubmit back.

[Note: HTML markup is below. Please do not edit.]

Reviewers' comments:

Reviewer's Responses to Questions

**Comments to the Author**

1. Is the manuscript technically sound, and do the data support the conclusions?

Reviewer #1: Yes

Reviewer #2: Yes

**********

2. Has the statistical analysis been performed appropriately and rigorously?

Reviewer #1: Yes

Reviewer #2: Yes

**********

3. Have the authors made all data underlying the findings in their manuscript fully available?

The PLOS Data policy

Reviewer #1: Yes

Reviewer #2: Yes

**********

4. Is the manuscript presented in an intelligible fashion and written in standard English?

Reviewer #1: Yes

Reviewer #2: Yes

**********

Reviewer #1: The manuscript presents a methodologically rigorous evaluation of two initiatives, and the data and analyses support the conclusions. The statistical approaches are applied carefully and transparently. The manuscript is well written and clear. However, some improvements will strengthen the manuscript, including: (1) clarifying access to third-party datasets in the Data Availability Statement, (2) specifying whether staff interviews required IRB review, (3) ensuring consistent definition and labeling of outcome variables and ATT units across the results and figures, and (4) more explicitly grounding the broader policy implications in the Discussion

Reviewer #2: Manuscript Title: Incentives are affective but insufficient to halt smallholder deforestation in the Colombia Amazon

Overall Assessment

The manuscript contributes to the increasing literature on impact evaluations of forests conservation policies by assessing and comparting two incentive-based initiatives implemented simultaneously at some extent in an specific context of the Colombian Amazon. The comparative perspective in this regard represents a valuable advance in this literature and could provide important insights to increase our understanding about what does (and does not) works in forest conservation. However, there are some important improvements that are required to be addressed before being accepted for publication. These are listed in the following:

1) My main concern is regarding the novelty of the research and its results. Although empirical results from the analysis are valuable and worthy; in the end, it reveals something that already has been widely discussed in the forest conservation literature: that incentive-based interventions, in most of the cases, yield positive yet modest deforestation outcomes. In this sense, I suggest you to discuss more explicitly across the manuscript (especially in the introductory and discussion Sections) what are the meaningful contributions that your analysis makes not only in terms of the empirical results that offer but also in relation to the theoretical and practical insights that could guide future research or practice in designing effective forest conservation policies.

2) Overall, the Methods Section is explain in great detail, however there are some methodological choice that seemingly are questionable. First, it is not clear enough whether grids used as controls cover only cover eligible areas (i.e farmers' managed lands) or did you the whole "impact analysis area" that, I suppose, include areas with differentiated tenure regimes (i.e. collective lands), or even, not defined property rights. If that is the case, this could imply that some unobserved differences were not completely controlled for. How did you deal with this potential issue?

3) Also, in relation to the methodological Section, while it is remarkable you apply a newer approach for testing the parallel trends assumption (PTA), in the interest of being transparent in regard to your results and their robustness, I would suggest that you could also perform and show the results of traditional either graphical or placebo PTA tests (at least as Supplementary Material).

4) Also, in this regard, would it be important to clarify whether and how did you control for spillovers in the evaluated outcome. Eventually, if you have not performed such an analysis, please elaborate the reasons of this decision. The analysis of potential spillovers could provide important insights in relation to the obtained results and potential mechanisms driving these.

5) It was not possible to combine existing socioeconomic data of the study area (maybe from primary or secondary sources) with the performed spatial analysis? Using this type of data to the analysis could bring important benefits to identify mechanisms driving difference in the interventions outcomes. In case this was not possible, at least needs to be listed as a potential limitation of the analyses and/or a potential future research.

6) In addition to criteria for targeting areas, was there any other difference in the design of analyzed intervention that might have enable difference in outcomes. Besides those discussed already, the shorter duration of the IFA contracts relative to longer and apparently more the intensive CTS treatment did not have implications in their overall lower deforestation impacts? Might these differences also have implications in terms of the permanence of their outcomes?

**********

what does this mean?). If published, this will include your full peer review and any attached files.

If you choose “no”, your identity will remain anonymous but your review may still be made public.

**Do you want your identity to be public for this peer review?**  For information about this choice, including consent withdrawal, please see our Privacy Policy

Reviewer #1: No

Reviewer #2: **Yes:**  Javier G. Montoya-Zumaeta

**********

[NOTE: If reviewer comments were submitted as an attachment file, they will be attached to this email and accessible via the submission site. Please log into your account, locate the manuscript record, and check for the action link "View Attachments". If this link does not appear, there are no attachment files.]

To ensure your figures meet our technical requirements, please review our figure guidelines: https://journals.plos.org/plosone/s/figures

You may also use PLOS’s free figure tool, NAAS, to help you prepare publication quality figures: https://journals.plos.org/plosone/s/figures#loc-tools-for-figure-preparation.

NAAS will assess whether your figures meet our technical requirements by comparing each figure against our figure specifications.

Attachment Submitted filename: PLOS one_rev comments.pdf

# Author comment: Author response to Decision Letter 1

**DOI:** 10.1371/journal.pone.0340539.r002

4 May 2026

Please see the Response to Reviewers file where we provide a detailed response to the Editor and the two Reviewers' comments

Attachment Submitted filename: Response to Reviewers.docx

# Peer review: Decision Letter 1

**DOI:** 10.1371/journal.pone.0340539.r003

**Authors:** Rajan Parajuli

18 May 2026

Incentives are effective but insufficient to halt smallholder deforestation in the Colombian Amazon

PONE-D-25-67913R1

Dear Dr. Corbera,

We’re pleased to inform you that your manuscript has been judged scientifically suitable for publication and will be formally accepted for publication once it meets all outstanding technical requirements.

Within one week, you’ll receive an e-mail detailing the required amendments. When these have been addressed, you’ll receive a formal acceptance letter and your manuscript will be scheduled for publication.

An invoice will be generated when your article is formally accepted. Please note, if your institution has a publishing partnership with PLOS and your article meets the relevant criteria, all or part of your publication costs will be covered. Please make sure your user information is up-to-date by logging into Editorial Manager at Editorial Manager® and clicking the ‘Update My Information' link at the top of the page. For questions related to billing, please contact billing support.

If your institution or institutions have a press office, please notify them about your upcoming paper to help maximize its impact. If they’ll be preparing press materials, please inform our press team as soon as possible -- no later than 48 hours after receiving the formal acceptance. Your manuscript will remain under strict press embargo until 2 pm Eastern Time on the date of publication. For more information, please contact onepress@plos.org.

Kind regards,

Rajan Parajuli

Academic Editor

PLOS One

Additional Editor Comments (optional):

Both reviewers found this version much improved with the all of their comments addressed. I also recommend accepting it.

Reviewers' comments:

Reviewer's Responses to Questions

**Comments to the Author**

Reviewer #1: All comments have been addressed

Reviewer #2: All comments have been addressed

**********

2. Is the manuscript technically sound, and do the data support the conclusions?

Reviewer #1: Yes

Reviewer #2: Yes

**********

3. Has the statistical analysis been performed appropriately and rigorously?

Reviewer #1: Yes

Reviewer #2: Yes

**********

4. Have the authors made all data underlying the findings in their manuscript fully available?

The PLOS Data policy

Reviewer #1: Yes

Reviewer #2: Yes

**********

5. Is the manuscript presented in an intelligible fashion and written in standard English?

Reviewer #1: Yes

Reviewer #2: Yes

**********

Reviewer #1: The revised manuscript is clear and strong. The authors have addressed my previous comments, especially by clarifying the study objectives, the logic behind comparing IFA and CTS, the treatment definitions, and the interpretation of the results. I also appreciate the added discussion of limitations. I recommend acceptance.

Reviewer #2: Thank you for addressing all my comments and suggestions. I think the paper provides a relevant contribution to the literature on PES and other incentive-based forest conservation initiatives.

**********

what does this mean?). If published, this will include your full peer review and any attached files.

If you choose “no”, your identity will remain anonymous but your review may still be made public.

**Do you want your identity to be public for this peer review?**  For information about this choice, including consent withdrawal, please see our Privacy Policy

Reviewer #1: **Yes:**  Ana Cubas-Baez

Reviewer #2: **Yes:**  Javier G. Montoya-Zumaeta

**********

# Editor report: Acceptance letter

**DOI:** 10.1371/journal.pone.0340539.r004

**Authors:** Rajan Parajuli

PONE-D-25-67913R1

PLOS One

Dear Dr. Corbera,

I'm pleased to inform you that your manuscript has been deemed suitable for publication in PLOS One. Congratulations! Your manuscript is now being handed over to our production team.

At this stage, our production department will prepare your paper for publication. This includes ensuring the following:

* All references, tables, and figures are properly cited

* All relevant supporting information is included in the manuscript submission,

* There are no issues that prevent the paper from being properly typeset

You will receive further instructions from the production team, including instructions on how to review your proof when it is ready. Please keep in mind that we are working through a large volume of accepted articles, so please give us a few days to review your paper and let you know the next and final steps.

Lastly, if your institution or institutions have a press office, please let them know about your upcoming paper now to help maximize its impact. If they'll be preparing press materials, please inform our press team within the next 48 hours. Your manuscript will remain under strict press embargo until 2 pm Eastern Time on the date of publication. For more information, please contact onepress@plos.org.

You will receive an invoice from PLOS for your publication fee after your manuscript has reached the completed accept phase. If you receive an email requesting payment before acceptance or for any other service, this may be a phishing scheme. Learn how to identify phishing emails and protect your accounts at https://explore.plos.org/phishing.

If we can help with anything else, please email us at customercare@plos.org.

Thank you for submitting your work to PLOS One and supporting open access.

Kind regards,

PLOS One Editorial Office Staff

on behalf of

Dr. Rajan Parajuli

Academic Editor

PLOS One
